# Network meta-analysis of transcriptome expression changes in different manifestations of dengue virus infection

**DOI:** 10.1186/s12864-022-08390-2

**Published:** 2022-02-27

**Authors:** Christine Winter, António A. R. Camarão, Imke Steffen, Klaus Jung

**Affiliations:** 1 Institute for Animal Breeding and Genetics, University of Veterinary Medicine Hannover, Bünteweg 17p, D-30559 Hannover, Germany; 2 Department of Biochemistry and Research Center for Emerging Infections and Zoonoses, University of Veterinary Medicine Hannover, Hannover, Germany

**Keywords:** Data fusion, Dengue fever, Network meta-analysis, Reproducibility, Robust data analysis, Transcriptome

## Abstract

**Background:**

Several studies have been performed to study transcriptome profiles after dengue virus infections with partly different results. Due to slightly different settings of the individual studies, different genes and enriched gene sets are reported in these studies. The main aim of this network meta-analysis was to aggregate a selection of these studies to identify genes and gene sets that are more generally associated with dengue virus infection, i.e. with less dependence on the individual study settings.

**Methods:**

We performed network meta-analysis by different approaches using publicly available gene expression data of five selected studies from the Gene Expression Omnibus database. The study network includes dengue fever (DF), hemorrhagic fever (DHF), shock syndrome (DSS) patients as well as convalescent and healthy control individuals. After data merging and missing value imputation, study-specific batch effects were removed. Pairwise differential expression analysis and subsequent gene-set enrichment analysis were performed between the five study groups. Furthermore, mutual information networks were derived from the top genes of each group comparison, and the separability between the three patient groups was studied by machine learning models.

**Results:**

From the 10 possible pairwise group comparisons in the study network, six genes (IFI27, TPX2, CDT1, DTL, KCTD14 and CDCA3) occur with a noticeable frequency among the top listed genes of each comparison. Thus, there is an increased evidence that these genes play a general role in dengue virus infections. IFI27 and TPX2 have also been highlighted in the context of dengue virus infection by other studies. A few of the identified gene sets from the network meta-analysis overlap with findings from the original studies. Mutual information networks yield additional genes for which the observed pairwise correlation is different between the patient groups. Machine learning analysis shows a moderate separability of samples from the DF, DHF and DSS groups (accuracy about 80%).

**Conclusions:**

Due to an increased sample size, the network meta-analysis could reveal additional genes which are called differentially expressed between the studied groups and that may help to better understand the molecular basis of this disease.

**Supplementary Information:**

The online version contains supplementary material available at 10.1186/s12864-022-08390-2.

## Background

Dengue is a mosquito-borne viral infection mainly distributed throughout the tropical and sub-tropical areas of the globe ([59]; https://apps.who.int/iris/handle/10665/44188). Being responsible for around 400 million infections every year it is the most prevalent arboviral disease of humans [2], and in recent years, its global incidence has grown dramatically [12]. Dengue is caused by four different dengue virus serotypes (DENV1–4) which are transmitted by infected female *Aedes aegypti* and *Ae. albopictus* mosquitoes [12]. According to the current World Health Organisation (WHO) criteria, dengue can be classified into (a) dengue with or without warning signs and (b) severe dengue. The clinical spectrum of dengue infections ranges from an unapparent subclinical picture to two well defined syndromes historically known as dengue fever (DF) and dengue hemorrhagic fever/dengue shock syndrome (DHF/DSS) [13]. DF is an acute self-limited systemic disease characterized by fever, headache, nausea, vomiting, myalgia, arthralgia, rash, and leukopenia. Individuals that may develop warning signs might present gingival bleeding, lethargy, hepatomegaly, thrombocytopenia and ascites and/or pleural effusion [59]. Ultimately, during the critical phase of infection the clinical picture may progress to a more severe form of disease characterized by capillary permeability and organ impairment [13]. Although virological and epidemiological risk factors contributing to the development of severe dengue have been identified, the molecular mechanisms underlying pathogenesis have yet to be characterized [13]. Nevertheless, it is noteworthy that for such a complex disease in its clinical manifestations the simple therapeutic management is highly effective in saving lives [60], as early detection of progression to the severe form of disease drops fatality rates of severe dengue to below 1% [35, 60]. The host transcriptional profile may be correlated with disease severity [15], hence, the characterization of the specific transcriptional patterns associated with each clinical form of disease could not only help anticipating the clinical progression and adequate management but also extend our knowledge on the molecular mechanisms underlying pathogenesis.

Transcriptome expression analyses by means of DNA microarrays [46] or next-generation sequencing (NGS) technology [32] can help to better understand the pathogenesis of virus infections as well as the role of genes and pathways in host responses [11, 17].

However, transcriptome expression data has a high-dimensional character, i.e. thousands of genes are studied in samples of usually small size, especially in cell line experiments [27]. Different techniques such as *p*-value correction have been proposed to reduce false positive findings [4], but transcriptome expression studies are often underpowered, increasing the chance of false negative results [22]. Therefore, several approaches for meta-analysis of transcriptome expression studies have been considered in order to achieve increased power and thus increased scientific evidence of findings [31, 41, 53]. In the context of infectious diseases, such methods have for example been applied for meta-analysis of transcriptome expression data from West Nile virus infected [20] or SARS-CoV2 infected samples [28]. Furthermore, meta-analysis can increase the reproducibility of gene expression analysis [49], and can increase the generalizability of results when data is taken from a variety of sources [10].

While meta-analysis as performed on clinical trials is usually done by two-stage approaches, i.e. results of individual analyses are merged by means of *p*-value- or effect-size-combination, meta-analysis of transcriptome expression data is often also possible in a single stage approach after merging the raw data of the involved studies. The single stage approach in the latter case is possible due to the free availability of high-dimensional gene expression data in public repositories such as Gene Expression Omnibus (GEO) [9] or ArrayEpress (AE) [6]. When merging gene expression data from independent studies additional steps for batch effect removal are usually necessary [14, 25].

In this meta-analysis on gene expression profiles from studies on dengue fever, we use the data merging approach which also allows to make group comparisons that were not performed in the original studies. This is obtained by building a network of study groups from the original data. We have previously shown that so called network meta-analysis on merged data (single-stage analysis) is highly correlated with network meta-analysis by means of two-stage analysis [56]. Network meta-analysis has first been presented in the context of clinical trials [45], and has also been used to mine protein expression databases [57]. Our analysis comprises differential expression analysis as well as gene ontology (GO) term enrichment analysis. We performed differential expression analysis once based on the merged data and once by a rank-based approach [19] on the results from the individual studies. The advantage of the merged data approach is an increased power but loses large numbers of genes in the merging step. The rank-based approach does not profit that much from an increased power but does not lose genes.

Using the top genes identified by both approaches, we derive mutual information networks to study changes in the pairwise correlation between genes, and fit machine learning models to the merged data to study the separability of different patient groups. Finally, we compare our new results with the results reported in the original studies.

## Results

### Database search and building of the study network

In March 2021, searches in the AE and GEO databases by the keyword ‘Dengue’ yielded 69 database entries, 31 of which referred to samples from *Homo sapiens*. Further selection left 29 datasets generated by either RNA-seq of coding RNA or by array-based transcription profiling. Because the samples of the 29 datasets were taken from different types of tissues, we manually screened the publications linked with the database entries. As result, 5 datasets remained where dengue fever was studied in whole blood samples (Table 1). All 5 datasets were generated by array-based transcription profiling. A flow diagram according to recommendations of the PRISMA guidelines for systematic reviews and meta-analyses [38] that depicts the selection process is provided as Supplementary Fig. S1. After outlier removal (see next subsection) 329 individuals remained from all groups.

**Table 1:** Selected data sets from NCBI GEO database and available sample sizes per study group, before and after outlier removal

Accession number	Healthy	Convalescent	DHF	DF	DSS	Reference
GSE13052		11			19	Long et al. [30]
GSE25226	4	6	6	20	8	Loke et al. [29]
GSE38246	8		32	53	20	Popper et al. [39]
GSE43777		24	37	40		Sun et al. [48]
GSE51808	9	19	10	18		Kwissa et al. [23]
Total	21	60	85	131	47	
Total after outlier removal	21	60	81	122	45	

**Fig. 1:**
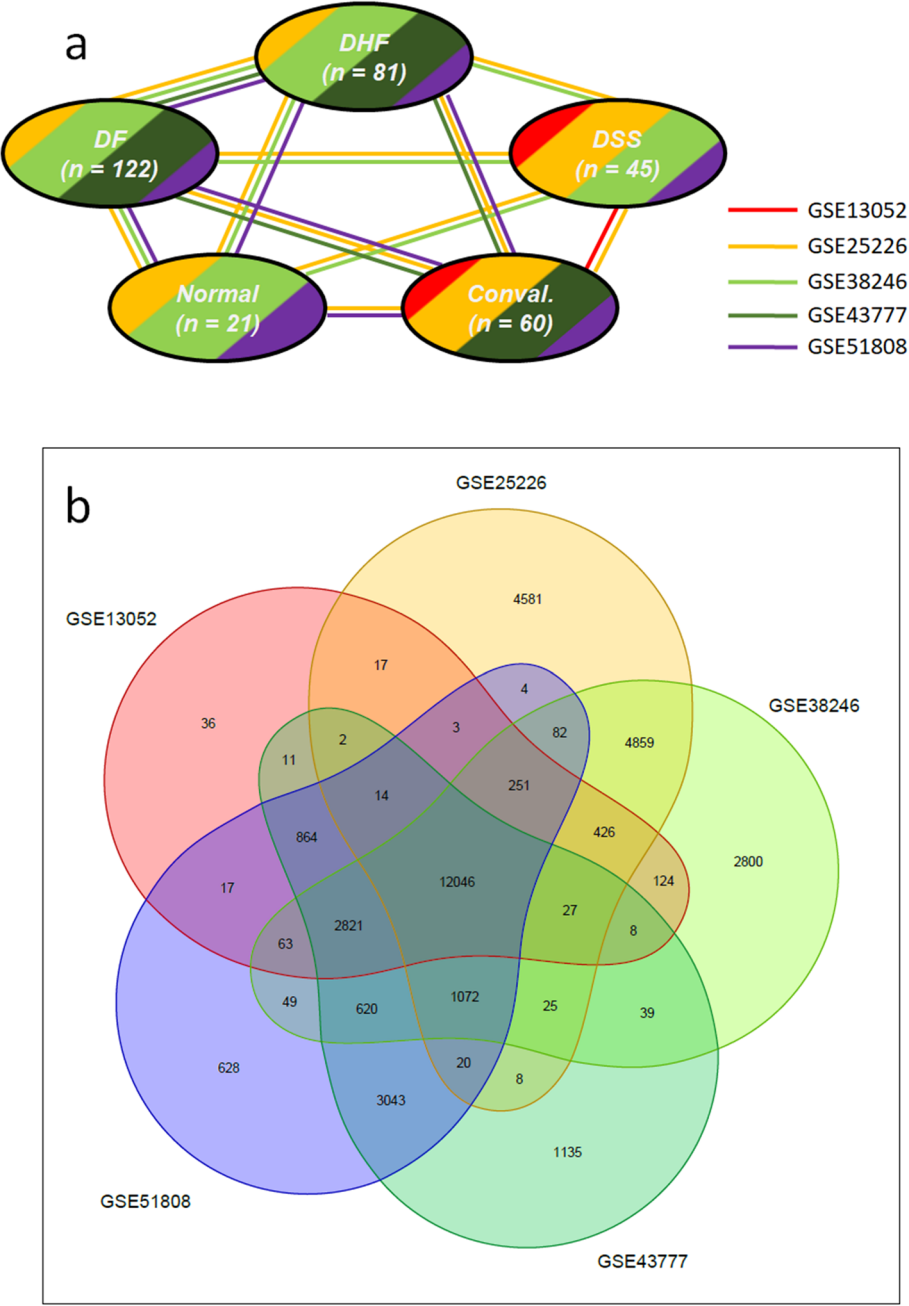
**A** Study network showing the availability and possible comparison of research groups in the five individual studies (labelled by GSE-accession numbers) as well as sample sizes per group summed over all studies. **B** Overlap of genes studied in the five individual studies. In total 12,046 genes were measured in all five studies

The resulting study network is illustrated in Fig. 1A, and is comprised of five different group: samples of 1) healthy control individuals, 2) of patients with dengue hemorrhagic fever (DHF), 3) of patients with dengue shock syndrome (DSS), 4) of patients with dengue fever (DF), and 5) of convalescent individuals. Only in the study by Loke et al. [29] data from all five groups was available in the databases. The other studies contributed with subsets of these five groups.

The studies by Long et al., Loke et al. and Popper et al. involved only children, while the studies by Sun et al. and by Kwissa et al. involved also adolescents and adults. The female/male ratio was in general well balanced, individual groups in some studies had slightly more males or females.

### Data merging, missing values imputation and batch effect removal

**Fig. 2:**
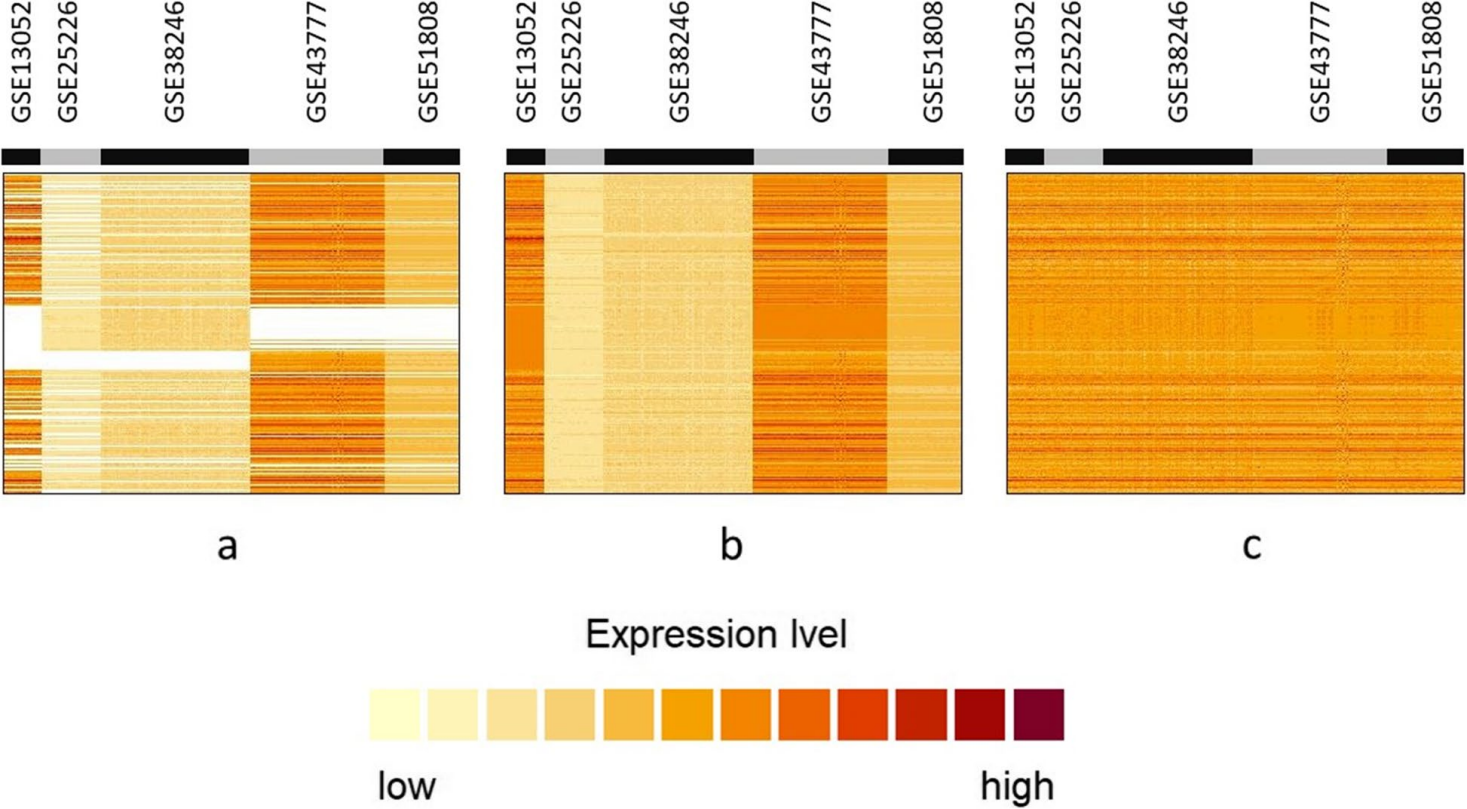
Unclustered images of transcriptome profiles from the five independent studies, **a** after data merging, **b** after missing values imputation by means of the *k*-nearest neighbour approach, and **c** after batch effect removal

In total, a union of 35.695 genes were involved in all five selected studies, with an intersect of only 12.046 that were studied in each of the studies (Fig. 1B). Before data merging, 9.180 genes which occurred in only one of the studies were removed from the data sets. Thus, only genes which occurred in at least two studies were considered for the merged data set which had a final size of 26.515 genes. The merged data matrix contained approximately 22% of empty entries which were filled by missing value imputation. Finally, batch effects, i.e. systematic differences between groups, were removed. The merging, imputation and batch effect removal steps are illustrated in Fig. 2. Note again, that the loss of genes concerns the differential expression analysis based on the merged data, but not the analyses based on rank aggregation.

**Fig. 3:**
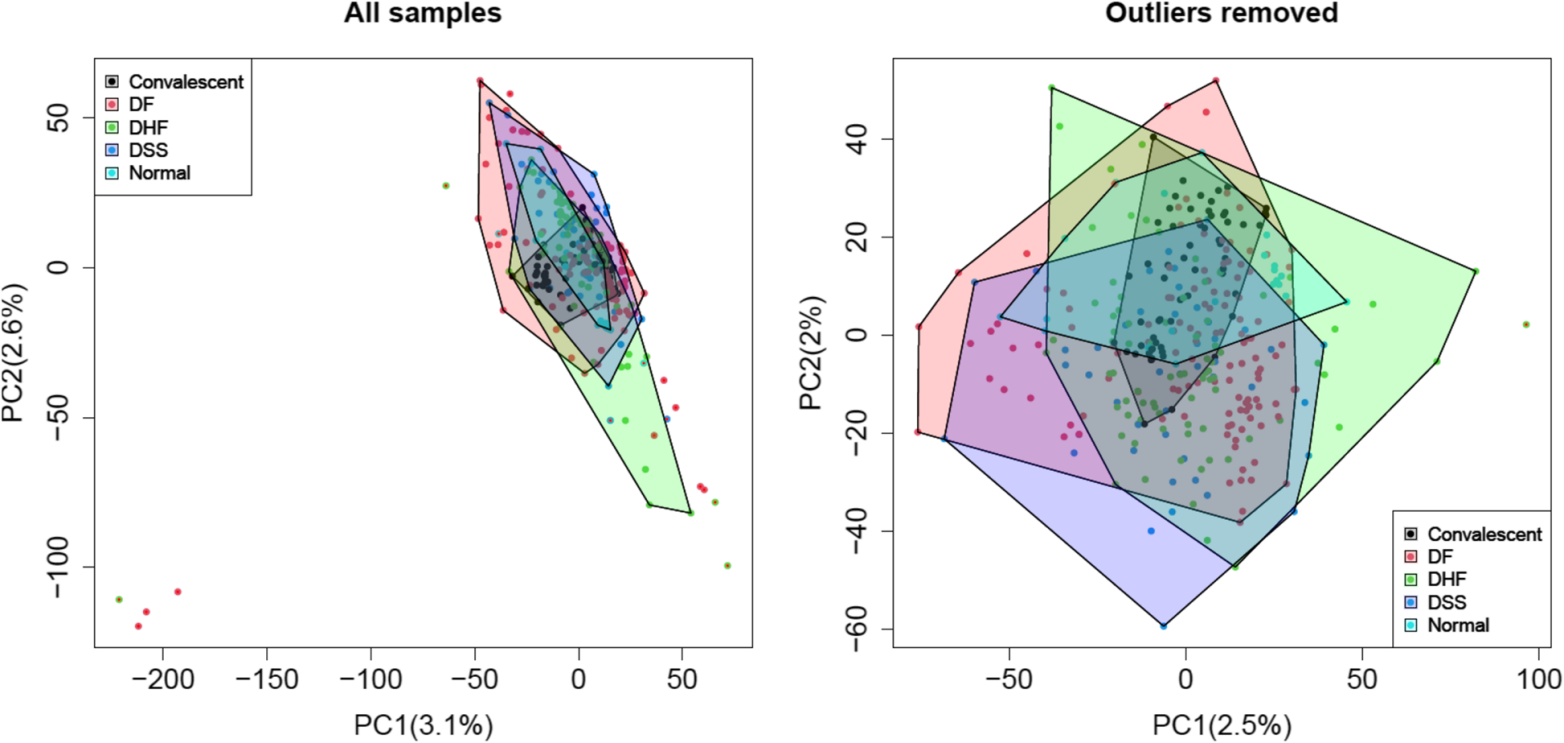
Left: principal component plot of all study groups, based on batch-effect adjusted data after data fusion. Right: new principal component plot after removal of outliers for the purpose of a robust analysis

Using group specific bag plots on the data projected to the space of the first two principal components, 15 outliers were detected in total and subsequently removed (Fig. 3) leading to a slightly reduced sample size. Four outliers were removed from the DHF group, 9 from the DF group and 2 from the DSS group (Table 1). Although, it is a statistically not recommended procedure to remove outliers from a data set just because these observations are extreme, we decided for our network meta-analysis to remove some outliers for several reasons. When fusing data from multiple independent studies there is a higher chance that some individuals may not belong to the particular disease group, and in consequence they do not fit the model well [43, 44]. Furthermore, it has been observed that in data mining single abnormal measurements might dominate the calculation of averages [5], which in the case of transcriptomics would lead to wrong estimates of the fold changes.

### Overview of results from differential gene expression

In this subsection, we report the results based on the merged data set. Additional results for differential expression analysis based on rank aggregation are reported in the subsequent subsection. As expected, the largest numbers of differentially expressed genes were found in the comparisons of the disease groups versus either the group of normal or versus the group of convalescent patients (Table 2). Taking only the FDR-adjusted *p*-values as selection criterion, the number varied between several hundred up to few thousand genes. The union of genes selected in the different group comparisons by an FDR-threshold of 5% were 7387 genes. For these genes, the p-values and log2 fold changes from all 10 pairwise group comparisons are provided as Supplementary Tables T1. When applying additionally a moderate threshold of +/− 1 for the log2 fold change, the numbers reduced clearly. In total, a union of 72 genes were found in all comparisons when using these strict selection criteria. According to these strict criteria (FDR-adjusted *p* < 0.05 & log2 fold change > +/− 1), no genes were selected when comparing DHF, DSS and DF groups among each other, or normal versus convalescent samples. A clustered heatmap based on these 72 genes and all 329 individuals is presented in Supplementary Fig. S2.

**Table 2:** Numbers of differentially expressed genes identified in the different pairwise group comparisons. Numbers in brackets are only based on FDR-adjusted *p*-values, the other numbers were obtained when using additionally a threshold for the log2 fold change. The last columns shows the numbers of significantly enriched GO-terms. Detailed results for each comparison for the union of 7387 genes and 111 GO terms are provided as Supplementary Tables T1 and T2

Comparison	# Differentially expressed genes	# Up-regulated	# Down-regulated	# Significantly enriched GO-terms
DF versus Normal	4 (582)	4 (421)	0 (161)	16
DHF versus Normal	10 (1378)	9 (957)	1 (421)	4
DSS versus Normal	23 (1921)	22 (1627)	1 (294)	24
Convalescent versus Normal	0 (0)	0 (0)	0 (0)	0
DHF versus DF	0 (23)	0 (0)	0 (23)	0
DSS versus DF	0 (1341)	0 (1180)	0 (161)	0
Convalescent versus DF	36 (2790)	35 (2043)	1 (747)	97
DSS versus DHF	0 (1072)	0 (961)	0 (111)	0
Convalescent versus DHF	51 (3489)	48 (2224)	3 (1265)	0
DSS versus Convalescent	67 (4972)	64 (4054)	3 (918)	35
Union	72 (7387)	68 (5794)	4 (1787)	111

For further analyses (such as the mutual information networks and classifier training) we used the ranking provided by the FDR-adjusted *p*-values without the log2 fold change as selection criterion. While a p-value comprises information about mean, variance and sample size, the fold change is only constructed using mean expression levels. In fact, a gene with a small fold change can still be significant if the within-group variances are small, while a gene with a large fold change can be non-significant if within-group variances are large. Therefore, significant genes can falsely be excluded by using a too strict fold change threshold.

### Top noticeable genes from differential expression analysis of the network meta-analysis

Among the different lists of top 10 genes from the two approaches of network meta-analysis (merged data analysis and analysis by rank aggregation), six genes occur more than three times (Table 3): IFI27 (7x), TPX2 (6x), CDT1 (5x), DTL (5x), KCTD14 (5x) and CDCA3 (4x). The high frequency of their occurrence among the top 10 lists provides evidence that these genes could play an important role in the context of DENV infections. IFI27, TPX2, CDT1 and CDCA3 are particularly known for their role in cancer [8, 36, 58, 61], and TPX2 also for its role in the context of infectious diseases, in particular dengue fever [51].

**Table 3:** Top 10 of differentially expressed genes from the pairwise comparisons in the study network. The first three columns result from the analysis of the merged data set, the last two columns results from the rank-based aggregation of differential expression analysis of the individual studies. The latter analysis does not yield p-values or fold changes but a score for each gene

**DF versus Normal**					**DHF versus Normal**				
Gene.ID	*p*	logfc	Name	Score	Gene.ID	*p*	logfc	Name	Score
IFI27	2,77E-13	1,94	OAZ2	1,05E−06	IFI27	1,91E-15	2,24	DAPK2	1,78E−06
GRAMD1C	3,81E-12	−0,7	TIGD3	1,09E−06	GRAMD1C	9,42E-12	−0,79	SIL1	2,25E-06
TIGD3	4,95E−12	−0,75	RNF141	1,62E−06	TIGD3	7,63E−08	− 0,66	KCTD14	2,72E− 06
CACNA2D3	5,80E-12	−0,96	TMCC1	1,89E−06	CACNA2D3	1,73E-10	-1,25	BUB1B	3,43E−06
CNTNAP3B	7,67E−10	-0,76	CABIN1	3,81E-06	CNTNAP3B	1,66E-07	-0,75	UBE2S	3,99E-06
STAP2	1,18E-09	0,47	LOC283588	4,16E-06	STAP2	1,58E-05	0,52	RGL2	6,12E-06
KCTD14	4,07E-09	0,99	CNTNAP3	4,34E-06	KCTD14	2,09E−10	0,91	CDC25A	9,55E-06
LOC283588	4,42E-09	-0,59	IFI27	7,72E-06	LOC283588	2,20E-08	-0,62	RNF141	9,55E-06
LINC00877	5,65E-09	-0,5	CD302	9,89E-06	LINC00877	4,02E-09	-0,56	MRPL40	1,04E-05
RNF141	6,49E-09	-0,39	MCM10	1,53E-05	RNF141	2,81E-07	-0,42	DTL	1,27E-05
**DSS versus Normal**					**DF versus Convalescent**				
Gene.ID	*p*	logfc	Name	Score	Gene.ID	*p*	logfc	Name	Score
KCTD14	4,75E−13	1,82	C6orf125	2,50E−05	IFI27	6,40E-30	3,07	TPX2	3,50E-10
IFI27	2,60E-12	2,31	MRPL22	0,00014352	DTL	2,73E-25	1,55	CDT1	6,79E−09
BARD1	8,54E-11	0,67	PTGS2	0,00015154	TPX2	3,40E-24	1,47	CDCA3	1,67E−08
MRPS18A	5,21E-10	0,58	CHMP1B	0,00017566	CDT1	5,50E-24	1,17	MKI67	1,40E−07
BIRC5	1,12E−09	1,03	FANCB	0,00018324	CACNA2D3	1,41E-23	-1,12	BAK1	4,05E−07
IFI27L1	1,60E− 09	0,99	HIST1H4K	0,00018324	CDC45	1,22E-22	1,09	KIF2C	4,50E−07
CDCA2	1,74E−09	1,1	LOC286052	0,00019229	CDCA3	1,13E-21	0,95	CDC20	6,62E− 07
CDCA8	2,01E−09	0,97	BARD1	0,00025716	C1QB	2,90E-21	1,25	DTL	8,39E−07
C16orf59	2,15E−09	0,6	IFI6	0,0002668	KIF2C	5,67E-21	0,99	CDC6	1,21E−06
MRPL22	2,48E−09	0,9	LOC285344	0,0002668	CDC20	5,94E-21	1,4	CHAF1A	2,08E−06
**DHF versus Convalescent**					**DSS versus Convalescent**				
Gene.ID	*p*	logfc	Name	Score	Gene.ID	*p*	logfc	Name	Score
IFI27	1,30E-26	3,37	TPX2	2,54E−09	CDT1	1,18E-20	1,33	TPX2	0,00011887
TPX2	1,50E-22	1,58	CDCA3	8,31E−09	TPX2	8,68E-20	1,63	OLIG1	0,00037903
CDT1	6,40E-21	1,14	DTL	1,26E-07	OLIG1	6,12E-19	-1,63	MRPS18A	0,00139727
CDCA3	6,60E-21	1,11	UHRF1	1,92E-07	MRPL22	6,19E-19	0,99	IFI27	0,00220204
CACNA2D3	9,60E-21	-1,42	CDC20	3,30E-07	IFI27	3,02E-18	3,44	NDUFA11	0,00220204
DTL	2,10E-20	1,53	KIF20A	3,56E-07	CDCA8	3,09E-18	1,19	DAD1	0,00440286
TK1	2,99E-20	1,24	CDKN3	5,49E-07	MRPS18A	8,69E-18	0,65	KCTD14	0,00440286
KIF2C	7,06E-19	1,08	CHEK1	7,57E-07	BARD1	3,70E-17	0,67	GLUL	0,00660247
MKI67	1,20E-18	1,22	CDT1	8,36E-07	CDCA2	6,90E-17	1,27	PTGDR2	0,00660247
CENPA	1,98E-18	0,84	RRM2	9,09E-07	TK1	7,06E-17	1,37	PSMB6	0,00774382
**DHF versus DF**					**DSS versus DF**				
Gene.ID	*p*	logfc	Name	Score	Gene.ID	*p*	logfc	Name	Score
ENPP5	3,22E-09	-0,46	PSKH2	3,94E-05	DNAJC15	7,73E-13	0,62	TRIP12	5,01E-05
NFE2L3	2,42E-07	-0,31	TRHR	4,57E-05	NME2	1,32E-12	0,43	DEFA4	0,0001334
ZNF675	3,75E-07	-0,3	CAMK2D	0,00013036	NELFE	4,72E-12	0,23	DNAJA1	0,0001334
RNF157-AS1	5,04E-07	-0,27	BTK	0,00019786	UQCRH	1,89E-11	0,5	LOC440474	0,00017441
LOC286087	6,10E-07	-0,52	TNF	0,00023865	PSMA6	5,85E-11	0,39	LOC283788	0,00019623
LOC158402	2,05E-06	-0,24	LOC153682	0,00024774	MDP1	6,15E-11	0,31	THBS1	0,00025415
CAMK2D	7,87E-06	-0,21	GAGE1	0,00028387	TMED7	7,01E-11	0,42	MKI67IP	0,0002668
LOC100128288	1,15E-05	-0,18	FIBCD1	0,00031375	SYVN1	8,75E-11	0,44	ROM1	0,0002668
SLC25A53	1,34E-05	-0,19	TRIM6	0,00032563	MTHFD1L	1,51E-10	0,44	FZD1	0,00029963
VPS41	1,54E-05	-0,19	LRP12	0,00035899	PSMA3	4,10E-10	0,52	PPFIBP2	0,00040019
**DSS versus DHF**					**Convalescent versus Normal**				
Gene.ID	*p*	logfc	Name	Score	Gene.ID	*p*	logfc	Name	Score
NME2	1,52E-11	0,41	CARD9	0,0001334	LOC442209	3,91E-06	-0,41	NCLN	0,00012672
PSMB2	1,52E-11	0,45	IFT80	0,0002668	SIGLECP3	5,39E-06	-0,48	KCNE3	0,00013053
KCTD14	3,38E-11	0,91	TMEM174	0,0002668	GOLGA8B	3,92E-05	-0,33	NFKBIE	0,00017469
NELFE	3,51E-11	0,21	BTLA	0,00040019	C8orf71	5,25E-05	0,28	ZNF20	0,00025344
PSME2	2,95E-10	0,3	LOC144742	0,00040019	LOC391766	5,84E-05	-0,34	ZNF586	0,00025344
C4A	8,16E-10	-0,44	BCL6	0,00053358	RPL26	6,11E-05	-0,49	KLHL7	0,0002853
PSMA6	9,14E-10	0,37	GDPD5	0,00053358	ANKRD30B	6,91E-05	-0,34	EIF5B	0,00038015
APOBEC3H	1,86E-09	0,36	RASSF4	0,00066697	LOC391040	7,23E-05	0,29	SNAI3	0,00038015
DNAJC15	4,16E-09	0,56	CD68	0,00080035	LOC283070	8,04E-05	-0,36	RUNX3	0,0004266
PSMA3	4,77E-09	0,48	LOC441309	0,00080035	LOC122423	0,00012596	0,32	SUSD1	0,00045673

In order to limit the findings not only to the top 10 lists, we also analysed the top 20 and top 50 lists. From all group comparison, 266 individual genes occur among the top 20, where 19 of them occur more than three times. If looking at the top 50, 618 individual genes are detected in total, where 48 of them occur more than 3 times. The additional 48 genes are listed in Supplementary Table T3. Still, the six above mentioned genes are those with the highest frequency among the top 20 and top 50 lists.

### Genes with strong correlation changes in mutual information networks

Here, we selectively describe mutual information networks with the largest changes between two groups. For each pair of study groups, we took the genes listed in Table 3, i.e. the top 10 from the merged data approach, and the top 10 from the rank aggregation approach. The union of these genes then had a size of ≤20 genes. The changes in the mutual information networks for all group comparisons are provided as Supplementary Figs. S3. While the presented Figure depict the changes in a mutual information network between two group, the group-specific mutual information adjacency matrices are presented as Supplementary Tables T4.

**Fig. 4:**
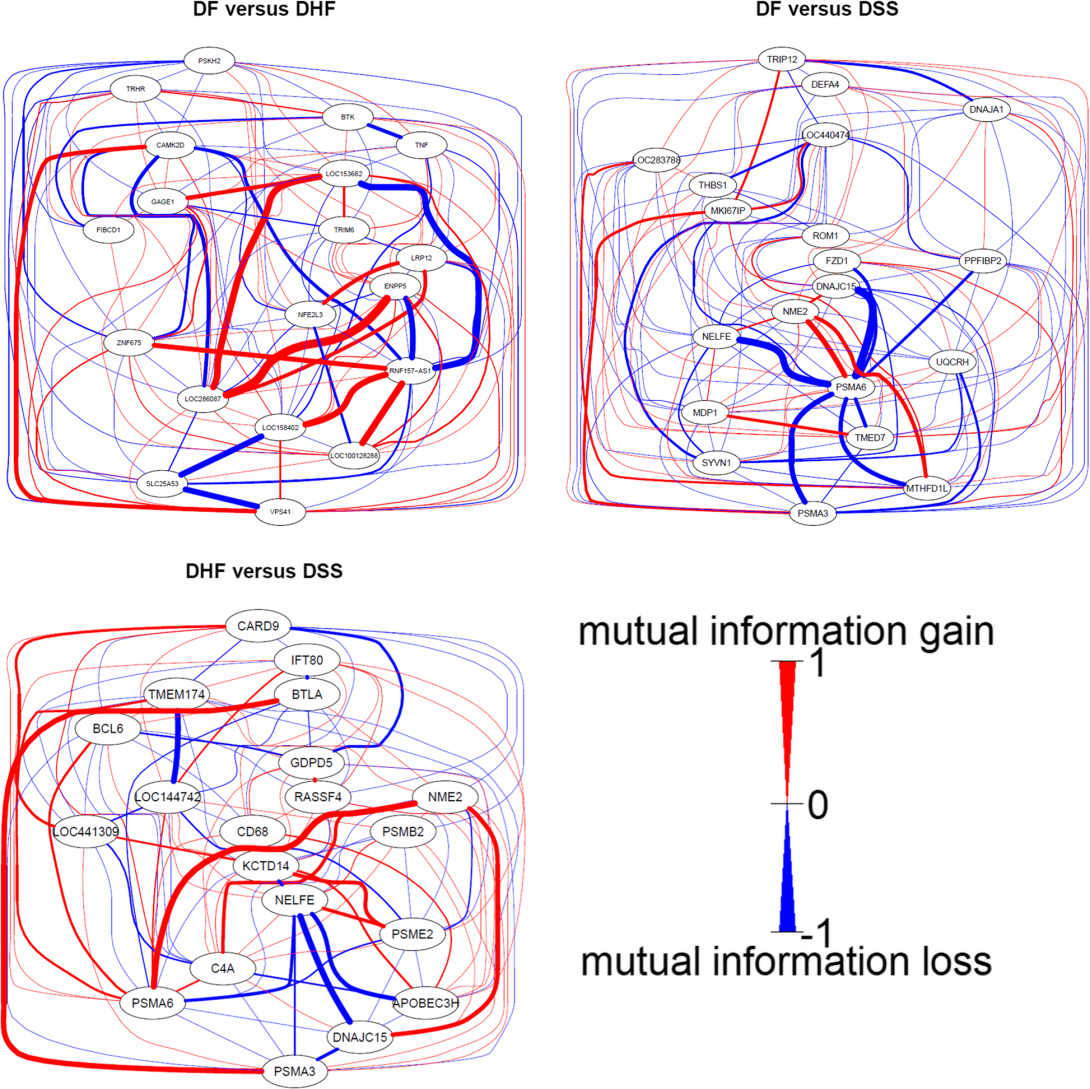
Mutual information networks based on top genes from each comparison between the three groups of patients, as identified from the network meta-analysis. Considering a comparison **A** versus **B**, blue edges indicate an increased correlation in samples from **A** compared to the correlation observed in **B**, while red edges indicate a stronger correlation in **B** compared to that in **A**. The thicker an edge, the stronger the observed correlation. Networks from the remaining seven comparisons are provided as Supplementary Material

In the mutual information network for DF versus DHF (Fig. 4) the correlation between LOC288086 and ENPP5 is strongly lowered in samples from DHF compared to the correlation in DF. Vice versa, the correlation between LOC153682 and RNF 157-AS1 is increased in DHF compared to DF.

In the network constructed from the comparison of DF versus DSS, the correlation of PSMA6 with NELFE and with DNAJC15 is stronger in DHF than in DF.

An increased correlation between NELFE and DNAJC15 in DSS samples is observed in the network constructed from the comparison of DHF versus DSS. In this network PSMA6 and NME2 lose their correlation in DSS samples compared to their expression in DHF samples.

### Gene ontology analysis

Table 2 also presents the number of significant GO terms from each comparison. Detailed results of the union of 111 selected GO-term analyses are also provided as Supplementary Tables T2. The most GO-terms were identified as significantly enriched in the comparisons with the normal group or with the group of convalescent patients. Again, no significant GO-terms were selected in the comparisons between DHF, DSS and DF group.

Among the significant GO terms are the general infection related terms such as “defense response to virus” (GO:0051607), “viral process” (GO:0016032) and “innate immune response” (GO:0045087). The latter two GO terms were also mentioned by the contributing study of Sun et al. [48]. Other overlapping GO terms with the results of Sun et al. are “mitotic spindle organization” (GO:0007052), “cell division” (GO:0051301), “mitotic spindle organization” (GO:0007052), “regulation of translation” (GO:0006417).

Among GO terms identified in the study by Loke et al. [29] was “nucleic acid binding” (GO:0003676). Our GO enrichment analysis identified the more specific terms “DNA binding” (GO:0003677) and “RNA binding” (GO:0005524) which are subcategories of “nucleic acid binding”.

### Separability of different dengue manifestations by transcriptome signatures

**Fig. 5:**
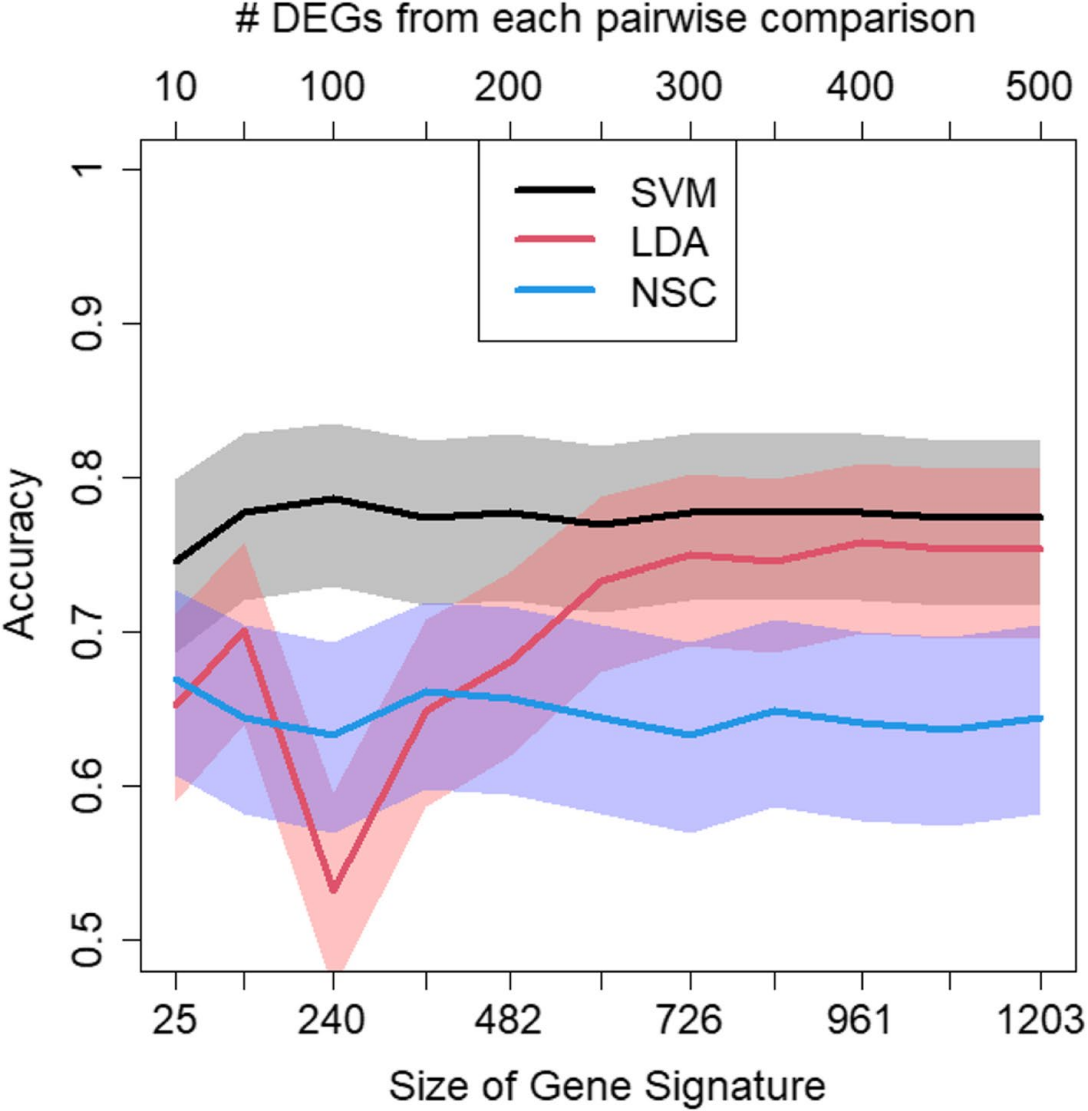
Accuracy and 95%-confidence bands in dependence of the number of genes involved in the classifier models for separating the dengue subtypes DF, DHF and DSS. The x-axis on the top represents the number of top ranked differentially expressed genes from each pairwise group comparison that contribute to each classifier. Due to the overlap of genes selected from each pairwise comparison, the final size of the signature (x-axis at the bottom) is smaller than 3 times the number given at the top. SVM = support vector machine; LDA = linear discriminant analysis; NSC = nearest shrunken centroids

Genes were ranked in each pairwise comparison between DF, DHF and DSS by their *p*-values. Classifier models were trained using unions of genes from each of the three ordered gene lists. Thus, if for example the top 10 genes of each of the three ranking lists were taken their union was ≤ 30 genes (note, that due to overlaps between three sets, the size of their union can be smaller than the sum of the individual sizes). At maximum, the 500 top ranked genes from each pairwise comparison were involved as predictors in each model. Due to overlaps, the maximum size of a classifier was given by 1203 genes (instead of 3*500 = 1500 genes). The final number of genes in each model is displayed on the x-axis at the bottom of Fig. 5. As can be seen, there, all classifier models yielded an accuracy bigger than 33.3% which would be achieved by a random classifier for three subtypes. The best performance was achieved by the support vector machine with which an accuracy of nearly 80% was obtained when taking approximately 250 genes as signature. Linear discriminant analysis yielded an accuracy of about 75%, however with 750 genes. Finally, nearest shrunken centroids yielded a nearly constant accuracy of about 67%, independently of how many genes were incorporated.

Looking at the individual groups, the DF group has the highest sensitivity. I.e., 117 out of 122 (96%) of DF patients were correctly classified by the support vector machine (with 250 genes) within cross vaidation as DF patients, while 1 DF patients was wrongly classified as DHF patients and 4 wrongly as DSS patient. The sensitivity of DHF was 60%, i.e. 49 out of 81 DHF patients were correctly classified while 31 DHF patients were classified as DF patients and 1 as DSS patient. Finally, 29 out of 45 (64%) of DSS patients were correctly classified the remaining 16 wrongly as DF patients. Thus, DHF and DSS patients tend to be wrongly classified as DF patients, and in consequence DF has a low positive predictive value. In cross validation, only 117 out of 164 (71%) patients classified as DF, where truly DF patients. In contrast, positive predictive values of DHF and DSS were 98% (49/50) and 85% (29/34), respectively. The individual curves of sensitivity, specificity, positive and negative predictive values versus the size of a classifier are provided as Supplementary Figs. S4.

## Discussion

### Methodical issues and reproducibility of findings from contributing studies

Performing meta-analyses of multiple independent studies has several benefits, foremost the increased power for statistical analysis. This is of special importance for high-throughput data where thousands of statistical tests bear the risk of false positive and false negative conclusions. In addition, the special character of a network meta-analysis facilitates indirect group comparisons that have not been performed in the individual studies. In the case of this work, each group comparison in the study network was directly performed by at least two of the individual studies. However, by using the data of all studies a particular group comparison can be supported by data from other studies. E.g., the comparison between DSS and convalescent individuals was only carried out in two studies originally. In the network meta-analysis by means of the merged data, all five studies contribute to this particular comparison.

Here, we have used two approaches for network meta-analysis, one based on the merged data and one rank-based approach. The advantages and disadvantages of either approach were given in the introduction. Besides, network meta-analysis can be performed using *p*-values, estimates of fold changes and their variances from the original data. Then, a new set of p-values and fold changes can be calculated by a graph theoretical framework [45]. While this framework avoids the problem of batch effect removal, inconsistencies within the network can occur. The chance of inconsistencies is of course increased in high-dimensional settings, where a network has to be built for each single gene.

Having different study settings or patient characteristics in the individual studies can be regarded as either an additional source for false conclusions, but also as an argument for producing results that are more generalizable. Here, different age groups contributed in the five original studies. Thus, one can argue that the genes we identified are more generally related to DENV infections, and do not depend on particular age groups.

When performing meta-analyses of high-dimensional data with the purpose of gene selection, there will be gains and losses in the new gene lists compared to those produced in the original studies. We don’t want to claim that the findings of the individual studies were false positive, just because there was little overlap with our new results. Besides the reason of different patient characteristics and sampling time points, the process of data merging and processing and the increased power can lead to different results. Also the perfect matching of gene lists by different aliases of gene names is still a problem for meta-analyses, not on the data level but on the text level of published manuscripts. Difficulties for meta-analysis also arise by incomplete or heterogeneously reporting of results in the original studies. Therefore, we tend to interpret our new findings rather as false negatives in the original studies, and suggest to incorporate the newly identified genes in the research on dengue infections.

### Reproducibility of findings from contributing studies

To further evaluate how the findings of the individual studies are in line with the findings of the network meta-analysis, we looked at the most important genes reported by these studies. In particular, we first compared our findings with individual genes highlighted in the manuscripts of the different publications. Loke et al. [29] mentioned 1525 genes in total that were differentially expressed between samples from DF, DHF and DSS individuals. Among these, the authors highlighted LTBR, PRAM1, CD14. None of these was found among the differentially expressed genes between the three patient classes in the network meta-analysis.

Among genes highlighted by Popper et al. [39] were Zinc finger genes, PLSCR1, ISG15, TBK1, TRIM25, H1F0, H3.3B, H2AFX, TOMM70A, c18orf22, WARS2, GLYCTK, GTPBP5, LYRM4, MTUS1, BCKDHB, ENOSF1, and SAMM50, ISG15, ISG20, OAS2, IFI27, STAT1 and STAT2, but only IFI27 was among the noticeable genes in the network meta-analysis.

Above, we already mentioned the signature reported by Sun et al. [48] included CACNA2D3 which was also found among the top genes in the network meta-analysis. Besides, their signature included reported LOC286087, SLC4A4, PSPH, MYOM2, CD244, SMAD5, where LOC286087 was also identified among the top genes in the network meta-analysis.

Kwissa et al. [23] highlighted CD16, CXCL-10,,CCL-2, CCL-4, IL1RN, IL-10, CCL11, IL-6, IL-8, CD206 (MMR), CD115 (M-CSFR), MCP-1 (CCL-2), IP-10 (CXCL-10), IL-6, IL-8, and IL-10, APRIL (TNFSF13) and BAFF, however, none of these were among the top genes of the network meta-analysis.

Besides genes highlighted in their manuscripts, Long et al. [30] present a supplementary list of top 100 selected genes (ranked by fold changes), Loke et al. [29] also present three supplementary lists of top 100 selected genes from the comparisons between DF, DHF and DSS groups, Popper et al. [39] present a supplementary list of about 300 genes from the different group comparisons, and Sun et al. [48] present two signatures (in total 142 genes) they identified from classifier analyses. Kwissa et al. [23] did not present additional tables. The overlap between these additional lists from the original studies and the union of the top 50 lists from the network meta-analysis are given in Supplementary Fig. S5. The overlap of reported genes among all of these reported lists was zero, even the overlap among all individual studies, showing a large heterogeneity of findings and little robustness. Nevertheless, some genes occurred as overlap between pairs, triplets and quadruplets of lists.

### Biological implications

As one of the mentioned six genes, and as a general noticeable player in infectious diseases, the function of IFI27 as part of the innate immune response in connection with viral diseases has also been reported in diverse organisms, including mice [24] and chicken [26]. Also in humans, the protein level increased after virus infection. Therefore, IFI27 expression was discussed as biomarker for viral infections [50]. This elevated expression is caused by various members of the flavivirus family. It has been associated with Zika, West Nile and dengue viruses infection [1, 40, 55]. During DENV infection, IFI27 is not only differentially expressed but further negatively correlated with the severity of the disease [37].

Except for DTL, the other five genes have been reported to distinguish between DSS and convalescent individuals by Long et al. [30] whose data also contribute to this meta-analysis. DTL itself has been shown to play role in oncogenic virus pathogenesis.

One gene that occurs only three times among the top 10 lists, CACNA2D3 has been reported by the contributing study of Sun et al. [48] as member of a molecular signature to distinguish between DHF and DF individuals. Besides, these mentioned genes have rarely been reported by the studies of the other contributing data sets, and could therefore be new candidates for further research on DENV infections.

The longitudinal characterization of the host transcriptome over time enables the assessment of the dynamic nature of the transcriptional profiles during the onset of the clinical disease upon DENV infection [48]. In line with this longitudinal study, others reported specific association of the transcript abundance pattern with the time course of disease [23, 39]. Generally, genes associated with innate immune sensing and pro-inflammatory responses to viral infection, in particular those encoding type I IFN-related proteins, cytokine/chemokine-mediated signaling and complement activity coincide with high viremia during the initial clinical disease phase [23, 48]. On the other hand, in a later phase of illness, genes associated with pathways involved in mitotic cell metabolic processes, translational control of protein biosynthesis and B cell differentiation are more prominent [29, 48]. In fact, the host transcriptional profile during DENV infections has been associated with the clinical manifestation/disease severity [29, 30, 39], time of sampling/day of illness [23, 48], viral load [23] and serological evidence of previous DENV exposure [39]. However, different studies favor certain features over others. For instance, in the study carried out by Kwissa and colleagues the transcriptional profile was not able to discriminate DF from DHF and was rather associated with the viral load than the clinical manifestation [23]. On the contrary, the differential transcriptome signatures observed between individuals with DF and DHF sampled on the same day after onset of disease suggests an association with the clinical manifestation rather than time of sampling [30]. Differentially expressed genes associated with a specific clinical manifestation were also described elsewhere, namely expression of mitochondrial ribosomal proteins was associated with DSS and genes encoding neutrophil-derived anti-microbial peptides were associated with DHF [29]. Remarkably, it is consistent that genes related with innate and adaptive immunity, such as IFN-mediated responses and antigen presentation and T cell priming respectively are down-regulated in individuals that develop severe dengue compared to those with uncomplicated disease [23, 39, 48]. Of particular importance in the context of severe dengue pathogenesis is the presumption that transcriptional events associated with capillary permeability and consequent hypovolemic shock may take place before cardiovascular collapse [30] which would have important prognostic potential.

## Conclusions

The meta-analysis of host genome-wide transcript expression profiles can be particularly valuable in the identification of candidate genes that may be used as biomarkers, supporting prognostic capacity and adequate clinical management in the context of DENV infection. Compared to the findings of the original studies, the findings of this meta-analysis may be more robust due to increased sample sizes in the individual groups. Nevertheless, we don’t argue that the new findings should replace the old once but rather complement. I.e., we assume that newly identified genes from the meta-analysis should be treated as false negatives in the original studies. As network meta-analyses have been very rarely performed on transcriptomics data, our study has also revealed methodical challenges and can provide ideas for methodical improvement.

## Methods

### Database search and study selection

Both repositories, AE and GEO, were queried using the search terms “Dengue”. For selection of studies, we followed the reporting criteria of the PRISMA statement [34] where possible. Inclusion criteria for the final selection were that samples of whole blood where taken from human individuals. We defined no selection exclusion criteria regarding age, gender or social or pathological variables.

### Data fusion and preparation for network meta-analysis

After determining a union of 35.695 genes that were in total regarded in the five studies a merged data set was built. After removing 9.180 genes that were solely present in only one of the studies, 26.515 genes remained for analysis. The *k*-nearest neighbour averaging method [54], implemented in the R-package ‘impute’, was used to impute missing values in the merged data set, i.e. for genes that were only present in two, three or four studies. Next, study specific batch effects were removed using the ComBat methods from the R-package ‘sva’ which implements the model of Johnson et al. [16]. This model accounts for study specific additive and multiplicative effects. In order to obtain more homogeneous study groups, outliers were removed using the bagplot approach on the principal components [21].

### Differential gene expression, mutual information networks gene ontology analysis

Pairwise group comparisons using the merged data set were performed with the R-package ‘limma’ to identify differentially expressed genes [47]. Resulting *p*-values were adjusted using the method of Benjamini and Hochberg to control for a false discovery rate of 5% [4]. In addition, confidence intervals for the log fold change were calculated [18]. In addition to the analysis of the merged data set, rank-based analysis based on the differential expression analysis of the individual data sets was performed using the R-packages ‘RobustRankAggreg’ [19].

The union of top10 genes from the merged-data-approach and the rank-based approach were subjected to analysis by mutual information networks with the R-package ‘minet’ [33], which themselves, were built on the merged data. Thus, each mutual information network was comprised by ≤20 genes. First mutual information networks of the selected genes were determined for each of the five study groups individually. For each network, the ‘minet’ function generates an adjacency matrix which stores the mutual information between each pair of genes in this network, normalized to values of the interval [0, 1]. In contrast to linear correlation, mutual information reflects more generally the difference between the joint distribution of two genes to the product of their marginal distributions. We visualized the results by overlapping the networks for each pair of study groups, so that changes in inter-gene-correlation between two study groups can be seen. In particular, the difference between the two adjacency matrices from two study groups was taken as basis of each network plot. Thus, gains and losses in the size of mutual information between two genes can be illustrated in the plots. The R-package ‘Rgraphviz’ was used to plot the networks.

Genes were annotated with gene ontology (GO) terms, and enrichment of GO terms among the differentially expressed genes was assessed by Fisher’s exact test [3]. GO annotations were taken from the UniProt database (www.uniprot.org).

### Training and evaluation of classifiers to separate different manifestations of dengue fever

Linear discriminant analysis [42], support vector machines [7] and nearest shrunken centroids [52] were used to train classifier models to study the separability of DF, DHF and DFF samples based on their expression profiles. Default settings of the R-packages ‘MASS’, ‘e1071’ and ‘pamr’ were used to fit the classifiers to the training data. As only hyperparameter, the number of genes involved in each transcriptome signature was additionally studied. Genes were selected from the lists of differentially expressed genes from all three pairwise comparisons between the three subtypes, and ranked by their raw *p*-values. Leave-one-out-cross validation was used to assess the accuracy of correct classification, as well as sensitivity, specificity and positive and negative predictive values.

## Supplementary Information

**Additional file 1: Supplementary Figure S1.** Flow diagram showing the selection process of transcriptome expression profiles from the ArrayExpression database.

**Additional file 2: Supplementary S2.** Heatmap of 72 selected genes.

**Additional file 3: Supplementary Figure S3.** Mutual information networks.

**Additional file 4: Supplementary Figure S4.** Sensitivity, specificity, positive and negative predictive values of classifier models.

**Additional file 5: Supplementary Figure S5.** Overlap of differentially expressed genes.

**Additional file 6: Supplementary Table T1.** Results from differentially expression analysis.

**Additional file 7: Supplementary Table T2.** Results from GO term enrichment analysis.

**Additional file 8: Supplementary Table T3.** Selection of 48 genes that occur frequently among the top 50, top 20, or top 10 of differentially expressed genes in the ten pairwise comparisons of the study network. The number give the absolute frequency, a gene occurs in one of the ten lists.

**Additional file 9: Supplementary Table T4.** Adjacency matrices from mutual information networks.

## Data Availability

The datasets analysed during the current study are available at GEO database: https://www.ncbi.nlm.nih.gov/geo/query/acc.cgi?acc=GSE13052, https://www.ncbi.nlm.nih.gov/geo/query/acc.cgi?acc=GSE25226, https://www.ncbi.nlm.nih.gov/geo/query/acc.cgi?acc=GSE38246, https://www.ncbi.nlm.nih.gov/geo/query/acc.cgi?acc=GSE43777, and https://www.ncbi.nlm.nih.gov/geo/query/acc.cgi?acc=GSE51808.

## References

[1] Azouz F, Arora K, Krause K, Nerurkar VR, Kumar M (2019). Integrated MicroRNA and mRNA profiling in zika virus-infected neurons. Viruses.

[2] Bhatt S, Gething PW, Brady OJ, Messina JP, Farlow AW, Moyes CL (2013). The global distribution and burden of dengue. Nature.

[3] Beißbarth T, Speed TP (2004). GOstat: find statistically overrepresented gene ontologies within a group of genes. Bioinformatics.

[4] Benjamini Y, Hochberg Y (1995). Controlling the false discovery rate: a practical and powerful approach to multiple testing. J Royal Stat Soc Ser B.

[5] Blessing RH (1997). Outlier Treatment in Data Merging. J Appl Crystallogr.

[6] Brazma A, Parkinson H, Sarkans U, Shojatalab M, Vilo J, Abeygunawardena N (2003). ArrayExpress—a public repository for microarray gene expression data at the EBI. Nucleic Acids Res.

[7] Chang CC, Lin CJ (2011). LIBSVM: a library for support vector machines. ACM Transact Intelligent Syst Rech.

[8] Cheriyath V, Leaman DW, Borden EC (2011). Emerging roles of FAM14 family members (G1P3/ISG 6–16 and ISG12/IFI27) in innate immunity and cancer. J Interf Cytokine Res.

[9] Edgar R, Domrachev M, Lash AE (2002). Gene expression omnibus: NCBI gene expression and hybridization array data repository. Nucleic Acids Res.

[10] Finfgeld-Connett D (2010). Generalizability and transferability of meta-synthesis research findings. J Adv Nurs.

[11] Gupta N, Rao PL (2011). Transcriptomic profile of host response in Japanese encephalitis virus infection. Virol J.

[12] Guzman MG, Harris E (2015). Dengue. Lancet.

[13] Halstead SB (2007). Dengue. Lancet.

[14] Hornung R, Boulesteix AL, Causeur D (2016). Combining location-and-scale batch effect adjustment with data cleaning by latent factor adjustment. BMC Bioinformatics.

[15] Jenner RG, Young RA (2005). Insights into host responses against pathogens from transcriptional profiling. Nat Rev Microbial.

[16] Johnson WE, Li C, Rabinovic A (2007). Adjusting batch effects in microarray expression data using empirical Bayes methods. Biostatistics.

[17] Josset L, Zeng H, Kelly SM, Tumpey TM, Katze MG (2014). Transcriptomic characterization of the novel avian-origin influenza a (H7N9) virus: specific host response and responses intermediate between avian (H5N1 and H7N7) and human (H3N2) viruses and implications for treatment options. MBio.

[18] Jung K, Friede T, Beißbarth T (2011). Reporting FDR analogous confidence intervals for the log fold change of differentially expressed genes. BMC Bioinformatics.

[19] Kolde R, Laur S, Adler P, Vilo J (2012). Robust rank aggregation for gene list integration and meta-analysis. Bioinformatics.

[20] Kosch R, Delarocque J, Claus P, Becker SC, Jung K (2018). Gene expression profiles in neurological tissues during West Nile virus infection: a critical meta-analysis. BMC Genomics.

[21] Kruppa J, Jung K (2017). Automated multigroup outlier identification in molecular high-throughput data using bagplots and gemplots. BMC Bioinformatics.

[22] Kurian SM, Whisenant T, Mas V, Heilman R, Abecassis M, Salomon DR (2017). Biomarker guidelines for high-dimensional genomic studies in transplantation: adding method to the madness. Transplantation.

[23] Kwissa M, Nakaya HI, Onlamoon N, Wrammert J, Villinger F, Perng GC (2014). Dengue virus infection induces expansion of a CD14+ CD16+ monocyte population that stimulates plasmablast differentiation. Cell Host Microbe.

[24] Labrada L, Liang XH, Zheng W, Johnston C, Levine B (2002). Age-dependent resistance to lethal alphavirus encephalitis in mice: analysis of gene expression in the central nervous system and identification of a novel interferon-inducible protective gene, mouse ISG12. J Virol.

[25] Leek JT, Johnson WE, Parker HS, Jaffe AE, Storey JD (2012). The sva package for removing batch effects and other unwanted variation in high-throughput experiments. Bioinformatics.

[26] Li X, Jia Y, Liu H, Wang X, Chu Z, Hu R, Yang Z (2019). High level expression of ISG12 (1) promotes cell apoptosis via mitochondrial-dependent pathway and so as to hinder Newcastle disease virus replication. Vet Microbiol.

[27] Lim WK, Mathuru AS (2020). Design, challenges, and the potential of transcriptomics to understand social behavior. Curr Zool.

[28] Liu H, Lin S, Ao X, Gong X, Liu C, Xu D, Ye H (2021). Meta-analysis of transcriptome datasets: an alternative method to study IL-6 regulation in coronavirus disease 2019. Comput Struct Biotechnol J.

[29] Loke PN, Hammond SN, Leung JM, Kim CC, Batra S, Rocha C, Harris E (2010). Gene expression patterns of dengue virus-infected children from Nicaragua reveal a distinct signature of increased metabolism. PLoS Negl Trop Dis.

[30] Long HT, Hibberd ML, Hien TT, Dung NM, Van Ngoc T, Farrar J (2009). Patterns of gene transcript abundance in the blood of children with severe or uncomplicated dengue highlight differences in disease evolution and host response to dengue virus infection. J Infect Dis.

[31] Marot G, Foulley JL, Mayer CD, Jaffrézic F (2009). Moderated effect size and P-value combinations for microarray meta-analyses. Bioinformatics.

[32] Metzker ML (2010). Sequencing technologies—the next generation. Nat Rev Genet.

[33] Meyer PE, Lafitte F, Bontempi G (2008). Minet: AR/bioconductor package for inferring large transcriptional networks using mutual information. BMC Bioinformatics.

[34] Moher D, Liberati A, Tetzlaff J, Altman DG (2009). Preferred reporting items for systematic reviews and meta-analyses: the PRISMA statement. Ann Intern Med.

[35] Moraes GH, de Fátima Duarte E, Duarte EC (2013). Determinants of mortality from severe dengue in Brazil: a population-based case-control study. Am J Trop Med Hyg.

[36] Neumayer G, Belzil C, Gruss OJ, Nguyen MD (2014). TPX2: of spindle assembly, DNA damage response, and cancer. Cell Mol Life Sci.

[37] Pandey AD, Goswami S, Shukla S, Das S, Ghosal S, Pal M (2017). Correlation of altered expression of a long non-coding RNA, NEAT1, in peripheral blood mononuclear cells with dengue disease progression. J Infect.

[38] Page MJ, McKenzie JE, Bossuyt PM, Boutron I, Hoffmann TC, Mulrow CD (2021). The PRISMA 2020 statement: an updated guideline for reporting systematic reviews. BMJ.

[39] Popper SJ, Gordon A, Liu M, Balmaseda A, Harris E, Relman DA (2012). Temporal dynamics of the transcriptional response to dengue virus infection in Nicaraguan children. PLoS Negl Trop Dis.

[40] Quicke KM, Suthar MS (2013). The innate immune playbook for restricting West Nile virus infection. Viruses.

[41] Rau A, Marot G, Jaffrézic F (2014). Differential meta-analysis of RNA-seq data from multiple studies. BMC Bioinformatics.

[42] Ripley BD. Pattern recognition and neural networks. Cambridge: Cambridge University Press; 2007. p. 91–120.

[43] Rousseeuw PJ, Hubert M (2011). Robust statistics for outlier detection. Wiley Interdiscip Rev.

[44] Rousseeuw PJ, Ruts I, Tukey JW (1999). The bagplot: a bivariate boxplot. Am Stat.

[45] Rücker G (2012). Network meta-analysis, electrical networks and graph theory. Res Synth Methods.

[46] Schena M, Shalon D, Davis RW, Brown PO (1995). Quantitative monitoring of gene expression patterns with a complementary DNA microarray. Science.

[47] Smyth GK (2005). Limma: linear models for microarray data. Bioinformatics and computational biology solutions using R and bioconductor.

[48] Sun P, García J, Comach G, Vahey MT, Wang Z, Forshey BM (2013). Sequential waves of gene expression in patients with clinically defined dengue illnesses reveal subtle disease phases and predict disease severity. PLoS Negl Trop Dis.

[49] Sweeney TE, Haynes WA, Vallania F, Ioannidis JP, Khatri P (2017). Methods to increase reproducibility in differential gene expression via meta-analysis. Nucleic Acids Res.

[50] Tang BM, Shojaei M, Parnell GP, Huang S, Nalos M, Teoh S, et al. A novel immune biomarker IFI27 discriminates between influenza and bacteria in patients with suspected respiratory infection. Eur Respir J. 2017;49(6): 1602098. 10.1183/13993003.02098-2016 28619954

[51] Tchankouo-Nguetcheu S, Khun H, Pincet L, Roux P, Bahut M, Huerre M (2010). Differential protein modulation in midguts of Aedes aegypti infected with chikungunya and dengue 2 viruses. PLoS One.

[52] Tibshirani R, Hastie T, Narasimhan B, Chu G. Class prediction by nearest shrunken centroids, with applications to DNA microarrays. Stat Sci. 2003;18(1):104–17.

[53] Tseng GC, Ghosh D, Feingold E (2012). Comprehensive literature review and statistical considerations for microarray meta-analysis. Nucleic Acids Res.

[54] Troyanskaya O, Cantor M, Sherlock G, Brown P, Hastie T, Tibshirani R (2001). Missing value estimation methods for DNA microarrays. Bioinformatics.

[55] Ubol S, Masrinoul P, Chaijaruwanich J, Kalayanarooj S, Charoensirisuthikul T, Kasisith J (2008). Differences in global gene expression in peripheral blood mononuclear cells indicate a significant role of the innate responses in progression of dengue fever but not dengue hemorrhagic fever. J Infect Dis.

[56] Winter C, Kosch R, Ludlow M, Osterhaus AD, Jung K (2019). Network meta-analysis correlates with analysis of merged independent transcriptome expression data. BMC Bioinformatics.

[57] Winter C, Jung K (2021). Mining protein expression databases using network Meta-analysis. Methods Molec Biol (Clifton, NJ).

[58] Wong AMG, Kong KL, Chen L, Liu M, Wong AMG, Zhu C, Guan XY (2013). Characterization of CACNA2D3 as a putative tumor suppressor gene in the development and progression of nasopharyngeal carcinoma. Int J Cancer.

[59] World Health Organization, Special Programme for Research, Training in Tropical Diseases, World Health Organization. Department of Control of Neglected Tropical Diseases, World Health Organization. Epidemic, & Pandemic Alert. (2009). Dengue: guidelines for diagnosis, treatment, prevention and control. Genf: World Health Organization; 2009.

[60] World Health Organization (2012). Global strategy for dengue prevention and control.

[61] Xouri G, Lygerou Z, Nishitani H, Pachnis V, Nurse P, Taraviras S (2004). Cdt1 and geminin are down-regulated upon cell cycle exit and are over-expressed in cancer-derived cell lines. Eur J Biochem.

