# Supplementary material for: Network meta-analysis of transcriptome expression changes in different manifestations of dengue virus infection
Source: BMC Genomics. 2022 Feb 27;23:165. doi: 10.1186/s12864-022-08390-2 (PMC8882220; doi:10.1186/s12864-022-08390-2)

Network meta-analysis of transcriptome expression changes in different manifestations of dengue virus infection

Christine Winter, António A. R. Camarão, Imke Steffen, Klaus Jung

Supplementary Figure: Flow diagram showing the selection process of transcriptome expression profiles from the ArrayExpression database.

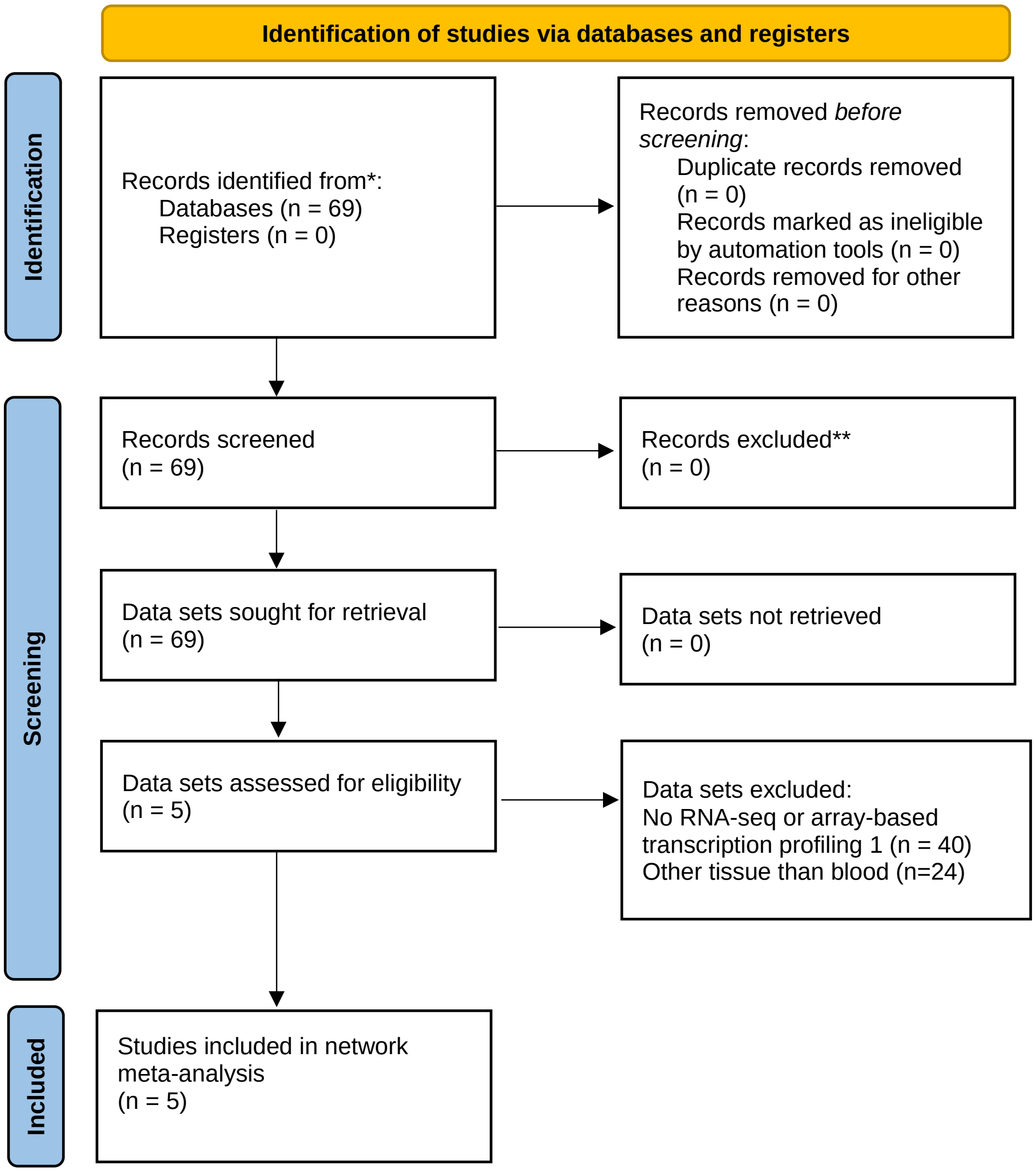

Supplement: Supplementary file 1 — Additional file 1: Supplementary Figure S1. Flow diagram showing the selection process of transcriptome expression profiles from the ArrayExpression database. [file 12864_2022_8390_MOESM1_ESM.pdf]
