# Supplementary figures and images for: Network meta-analysis of transcriptome expression changes in different manifestations of dengue virus infection
Source: BMC Genomics. 2022 Feb 27;23:165. doi: 10.1186/s12864-022-08390-2 (PMC8882220; doi:10.1186/s12864-022-08390-2)

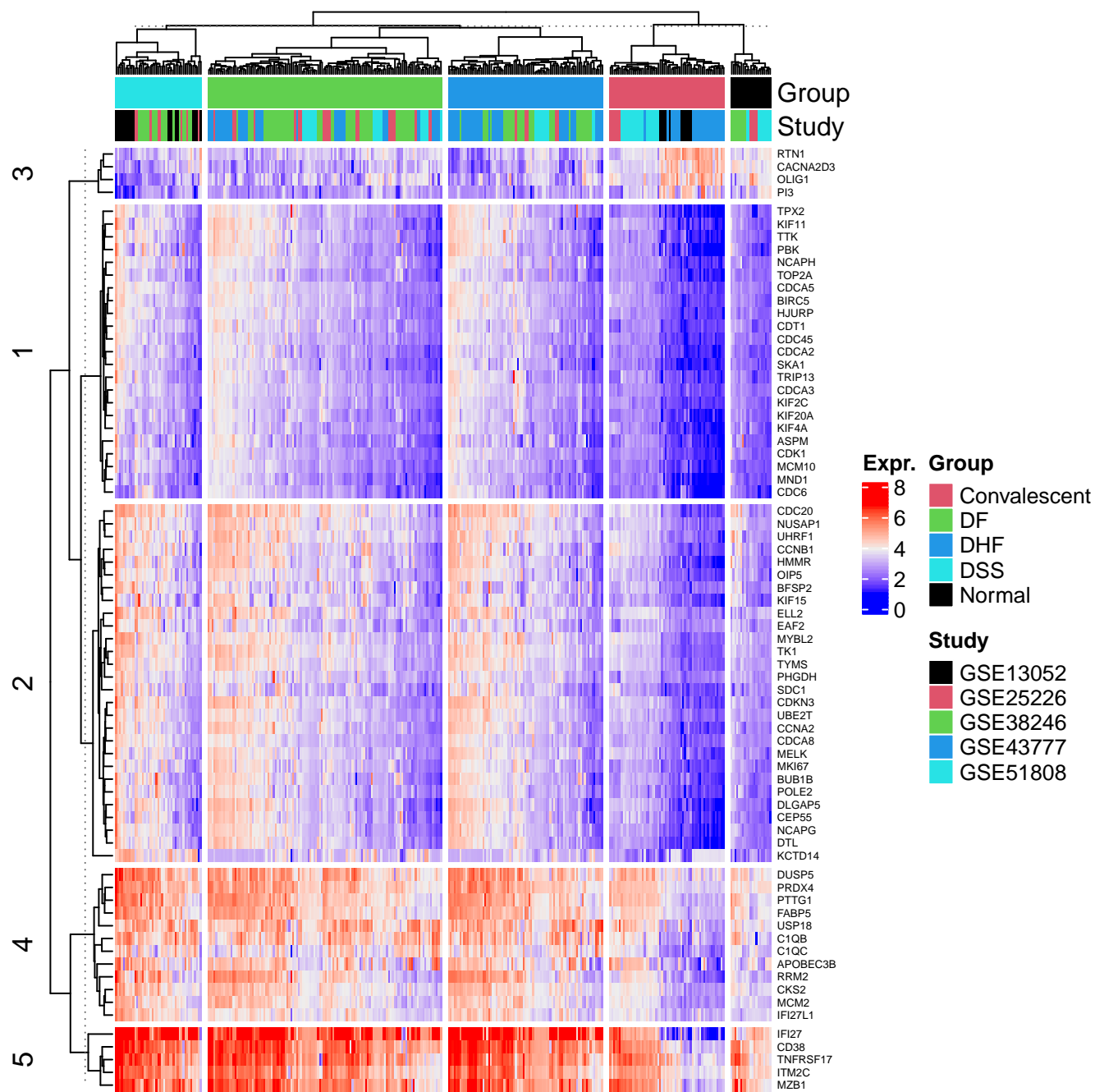

Supplement: Supplementary file 2 — Additional file 2: Supplementary S2. Heatmap of 72 selected genes. [file 12864_2022_8390_MOESM2_ESM.pdf]
