# Supplementary material for: Network meta-analysis of transcriptome expression changes in different manifestations of dengue virus infection
Source: BMC Genomics. 2022 Feb 27;23:165. doi: 10.1186/s12864-022-08390-2 (PMC8882220; doi:10.1186/s12864-022-08390-2)

## Slide 1
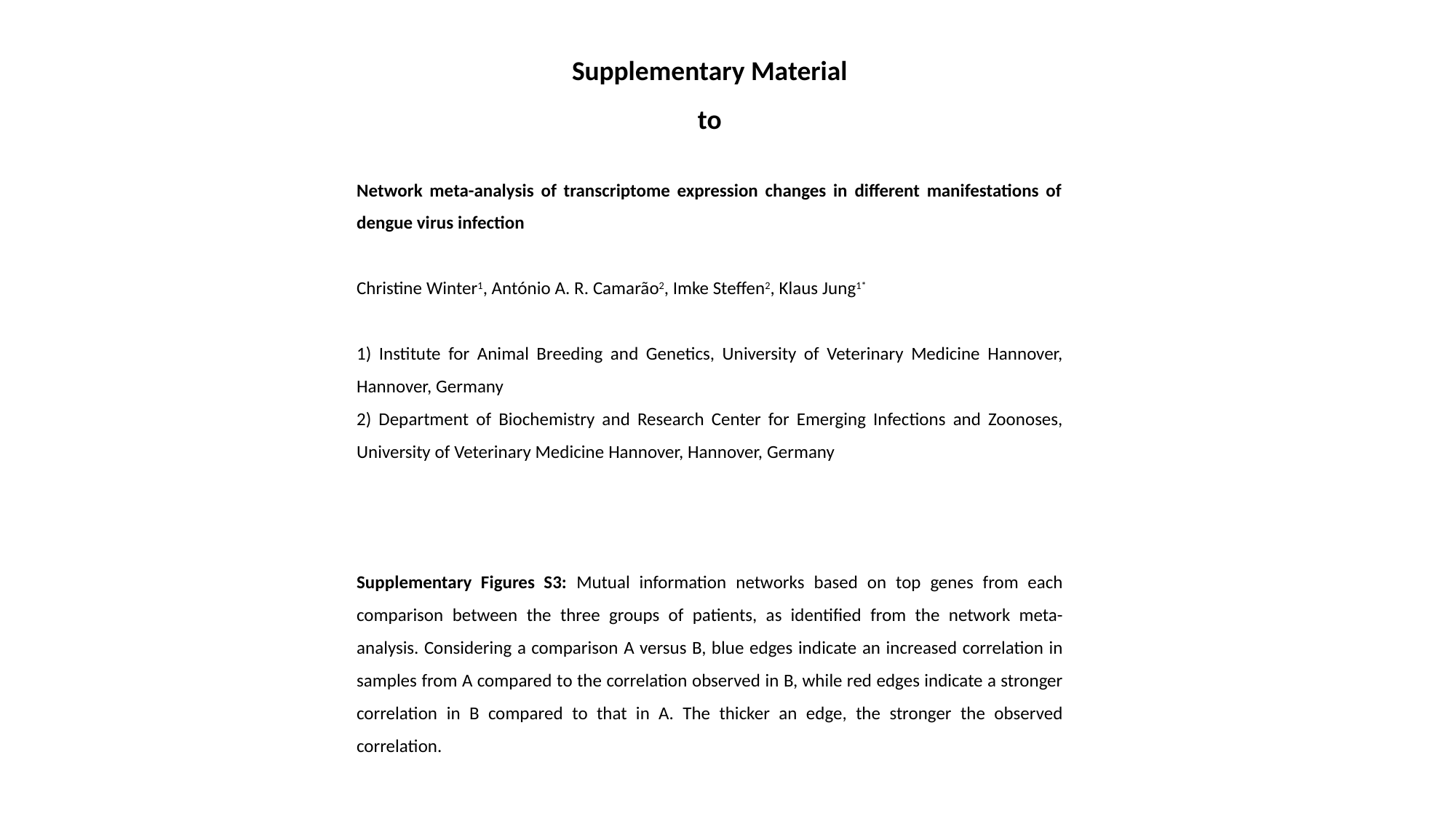

Supplementary Material
to
Network meta-analysis of transcriptome expression changes in different manifestations of dengue virus infection
Christine Winter1, António A. R. Camarão2, Imke Steffen2, Klaus Jung1*
1) Institute for Animal Breeding and Genetics, University of Veterinary Medicine Hannover, Hannover, Germany
2) Department of Biochemistry and Research Center for Emerging Infections and Zoonoses, University of Veterinary Medicine Hannover, Hannover, Germany
Supplementary Figures S3: Mutual information networks based on top genes from each comparison between the three groups of patients, as identified from the network meta-analysis. Considering a comparison A versus B, blue edges indicate an increased correlation in samples from A compared to the correlation observed in B, while red edges indicate a stronger correlation in B compared to that in A. The thicker an edge, the stronger the observed correlation.

## Slide 2
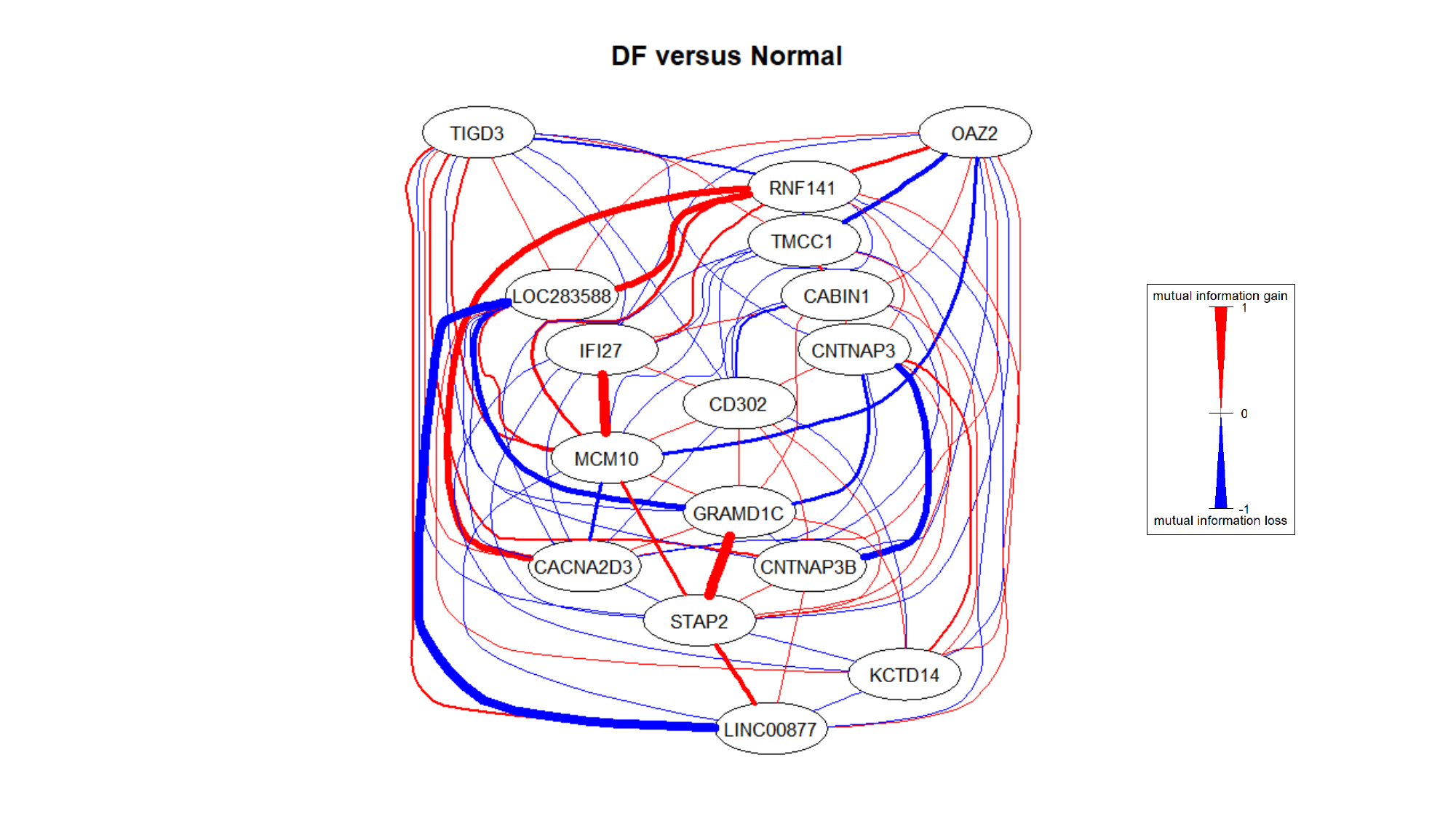

## Slide 3
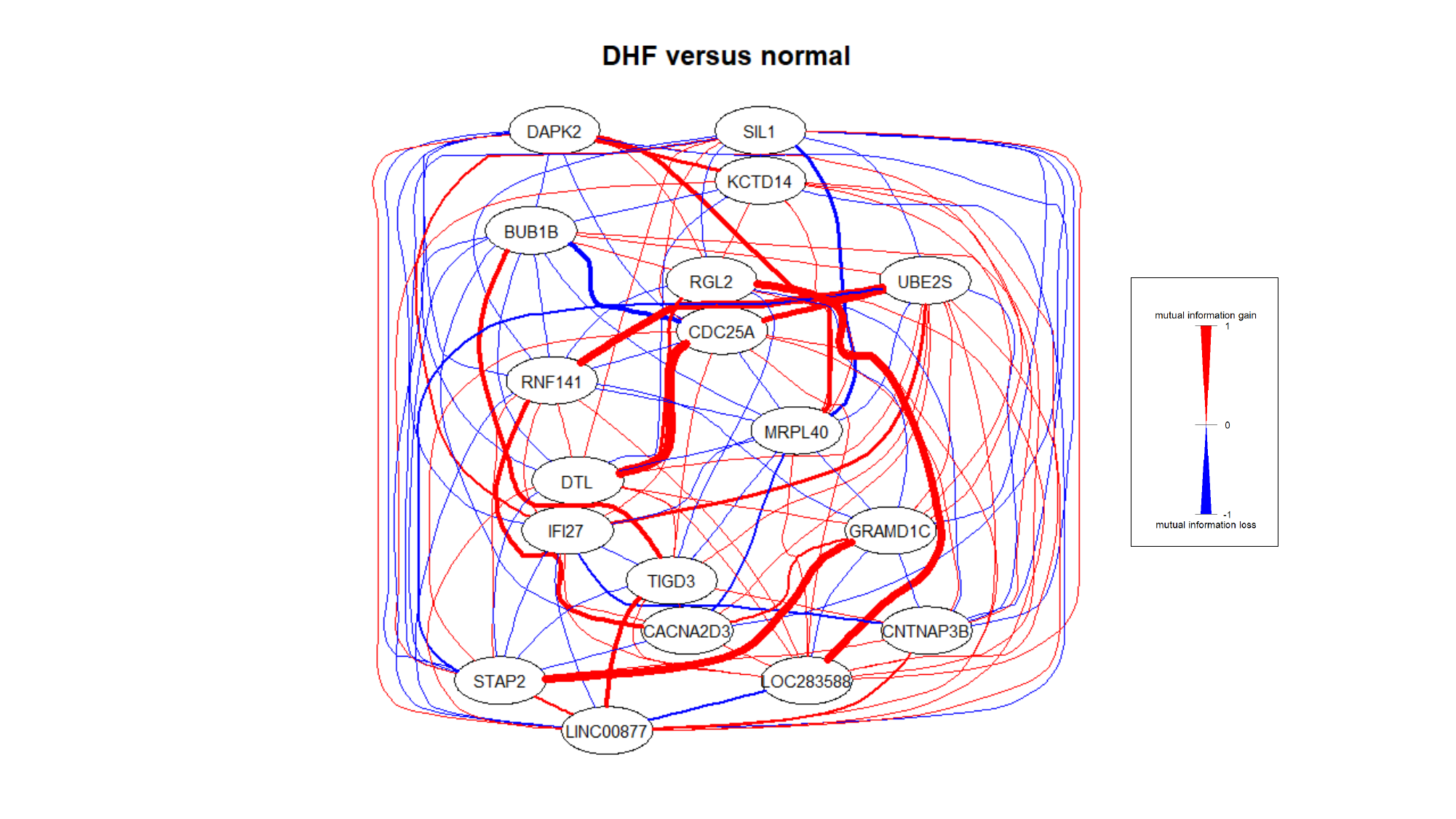

## Slide 4
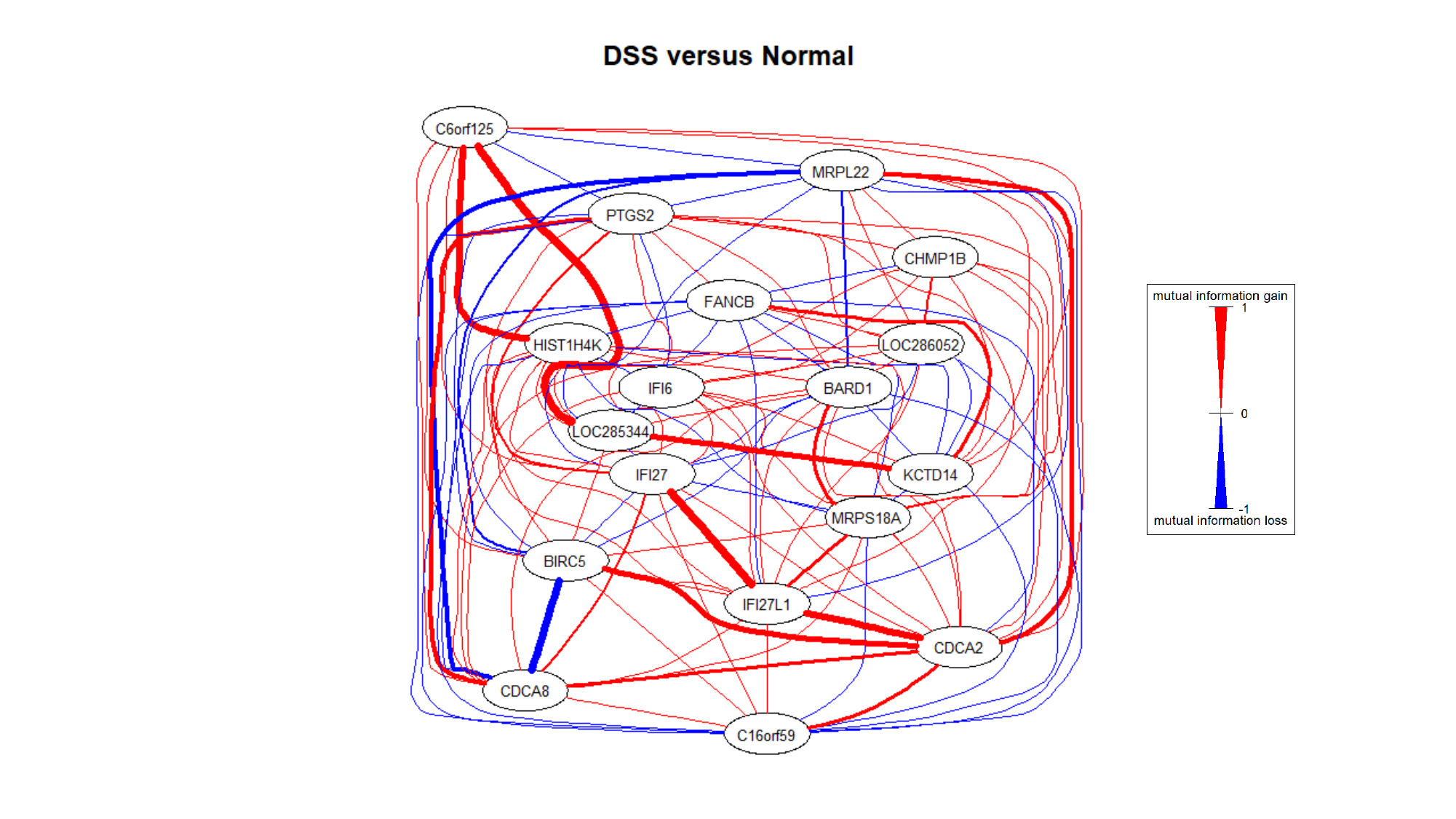

## Slide 5
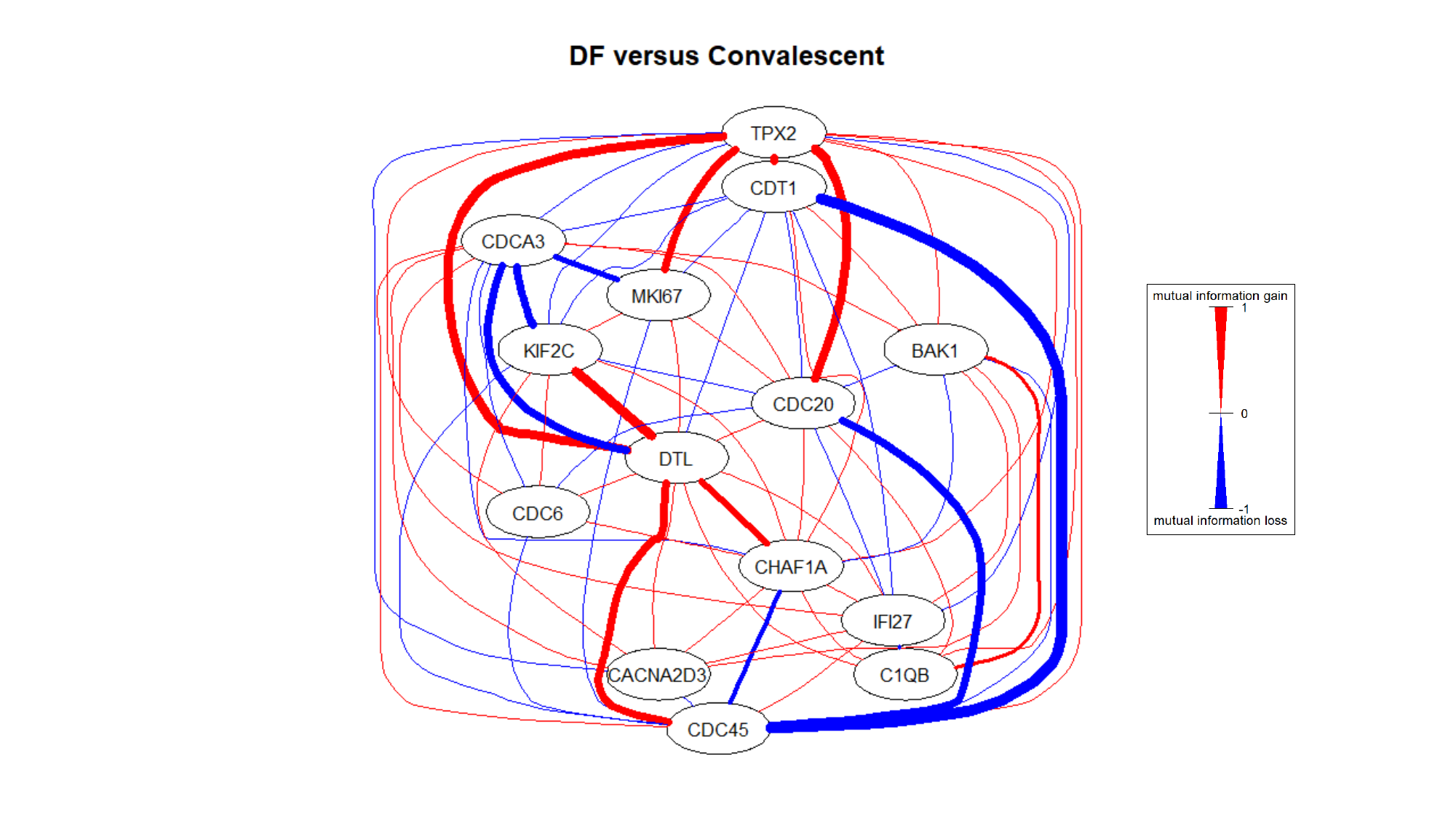

## Slide 6
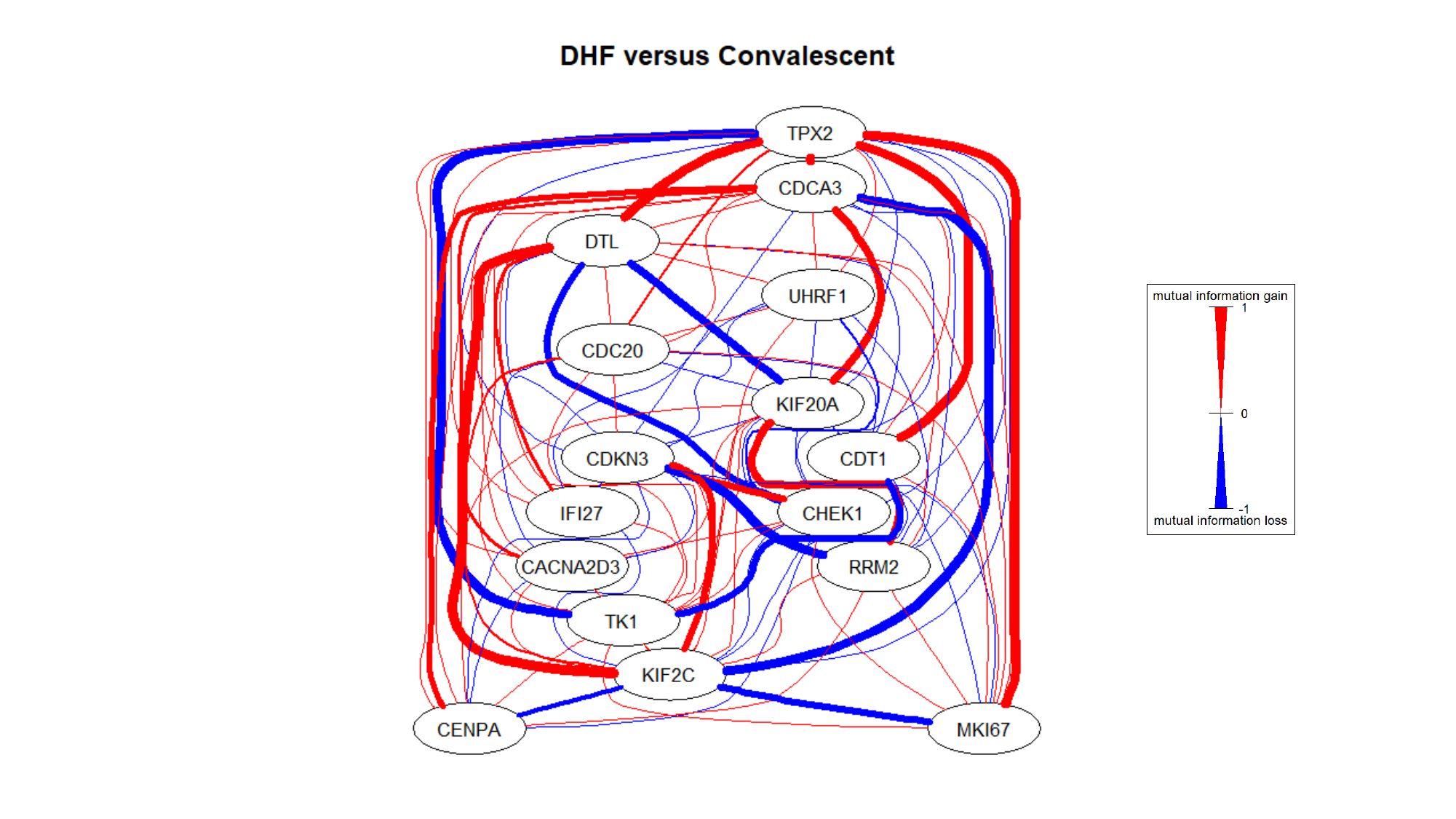

## Slide 7
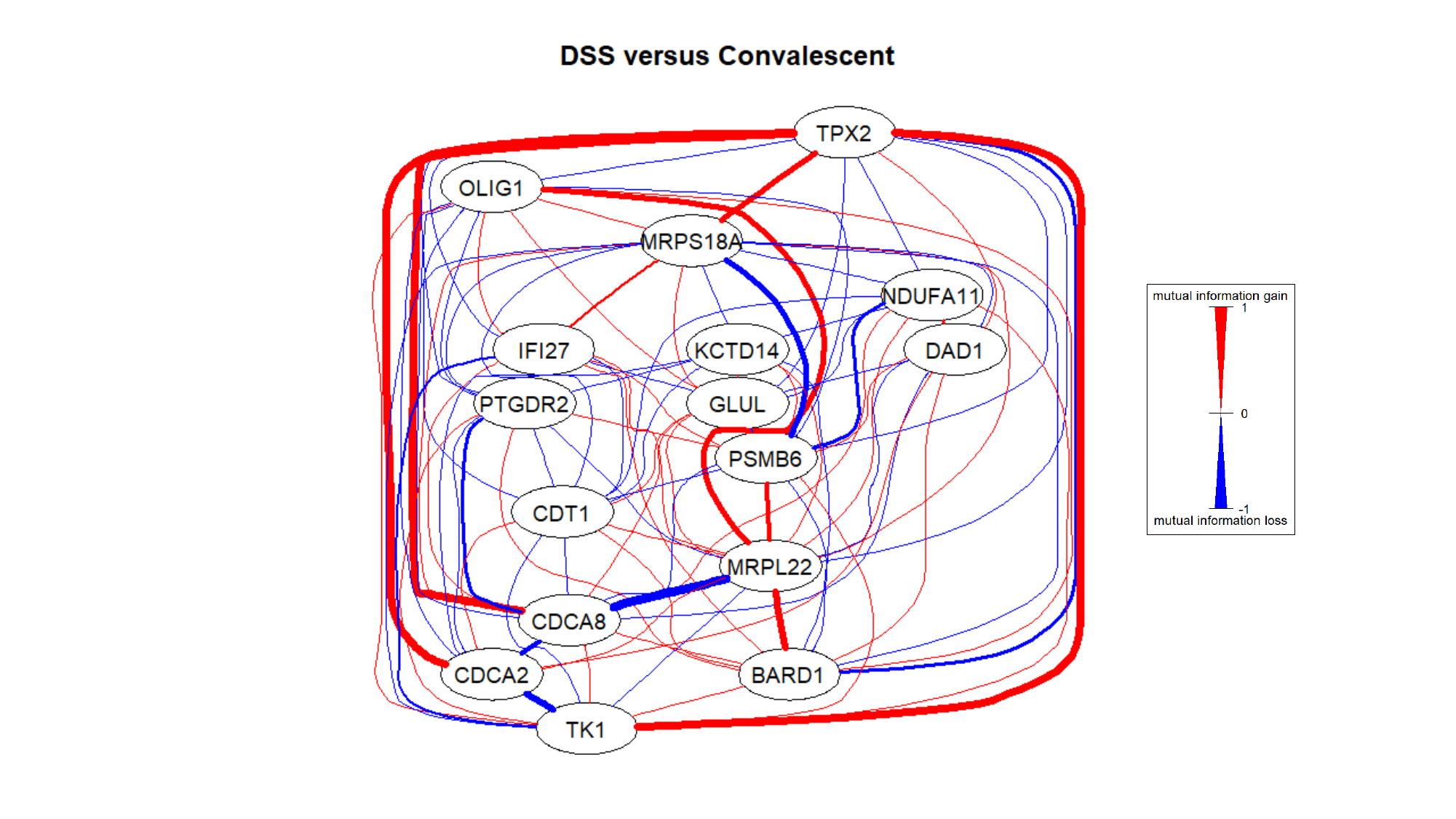

## Slide 8
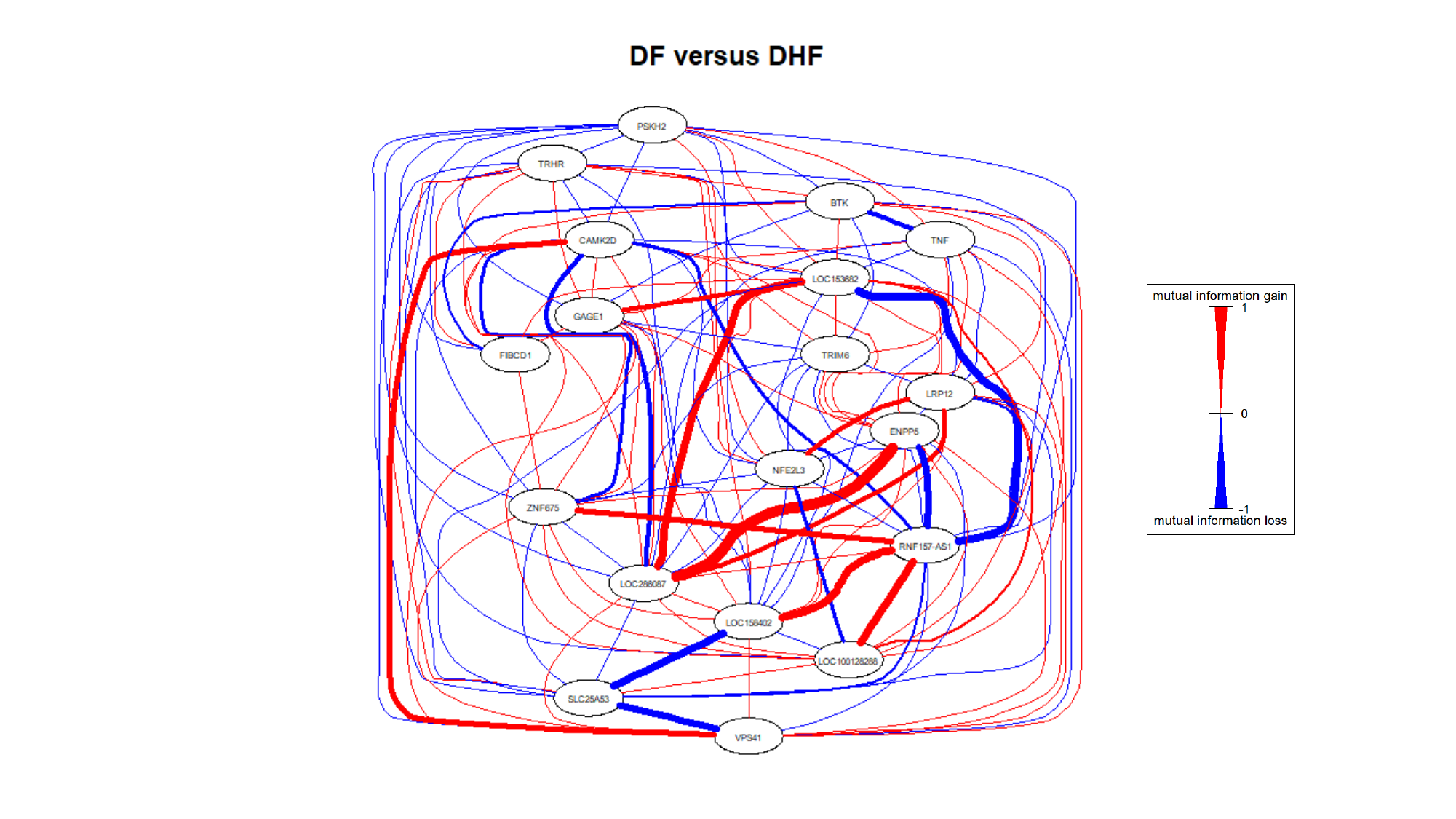

## Slide 9
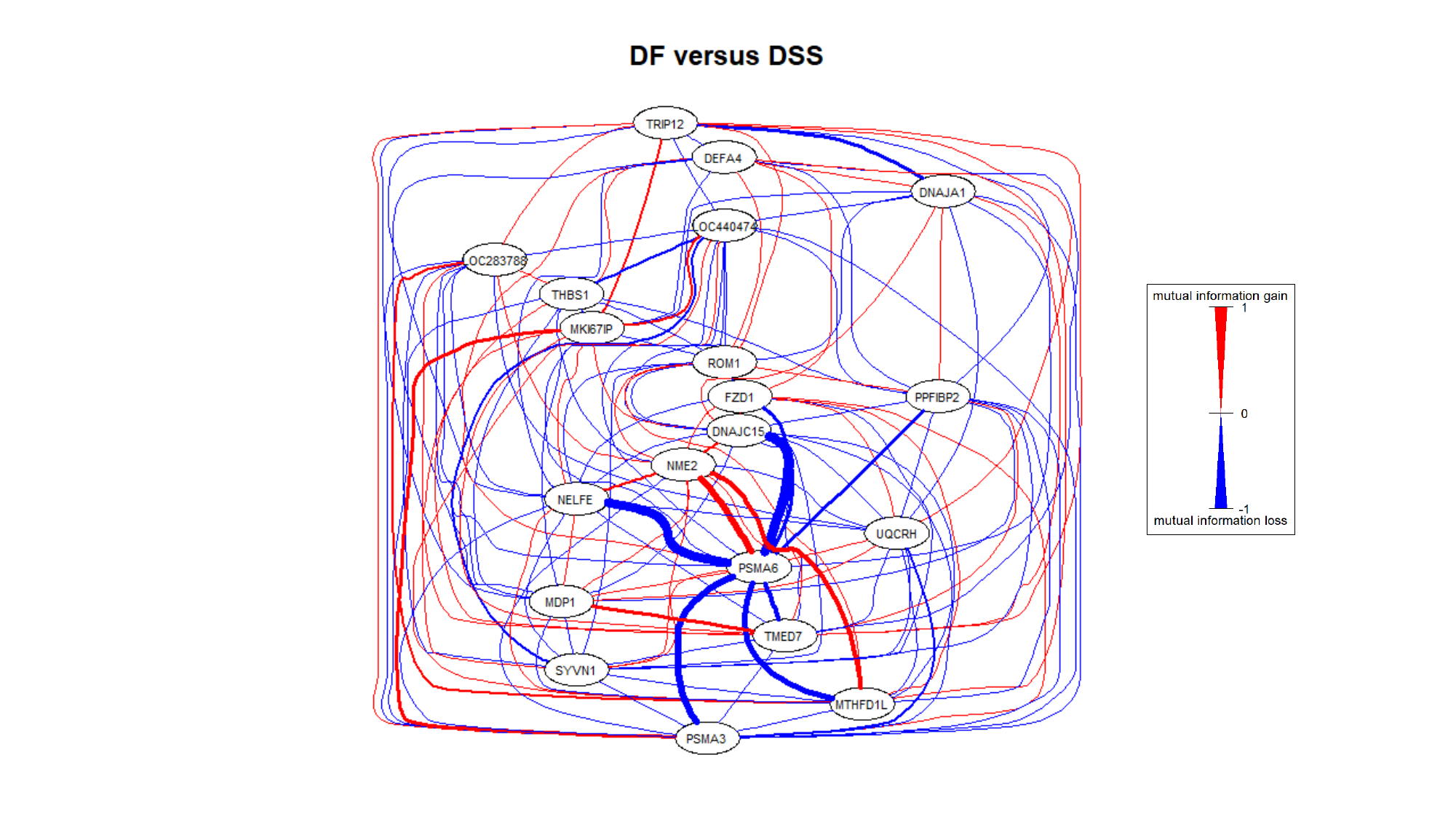

## Slide 10
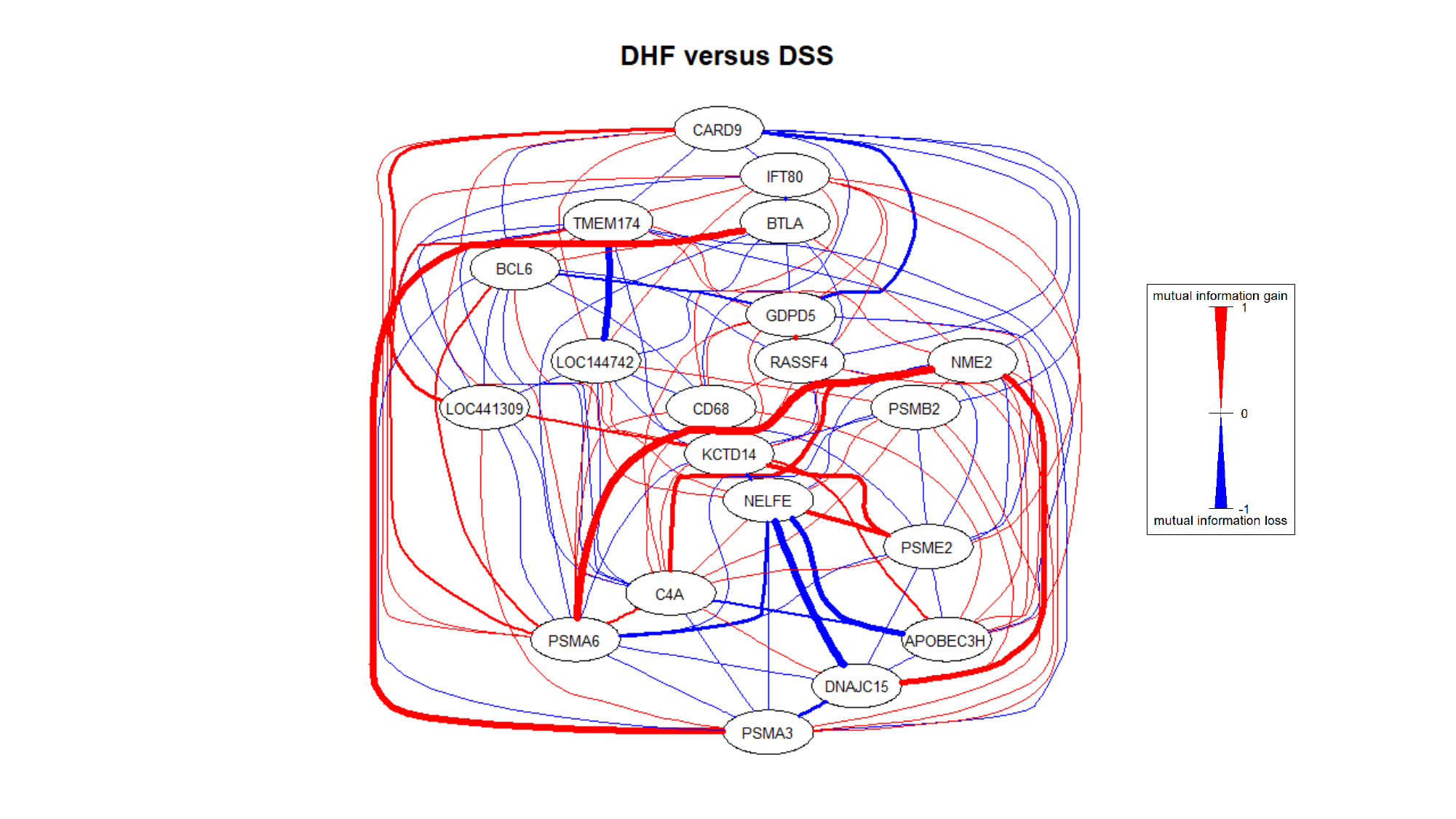

## Slide 11
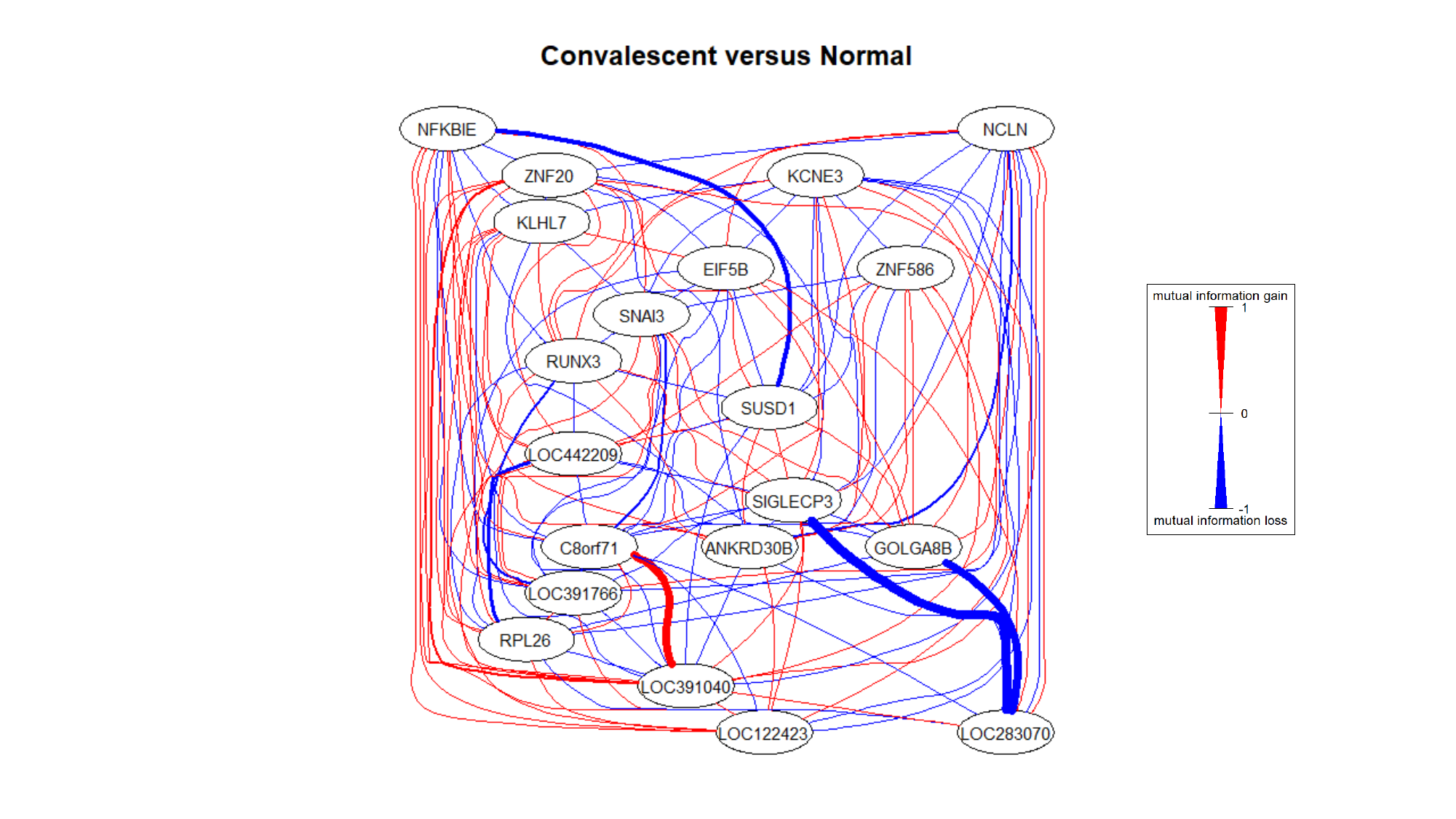

Supplement: Supplementary file 3 — Additional file 3: Supplementary Figure S3. Mutual information networks. [file 12864_2022_8390_MOESM3_ESM.pptx]
