# Supplementary material for: Network meta-analysis of transcriptome expression changes in different manifestations of dengue virus infection
Source: BMC Genomics. 2022 Feb 27;23:165. doi: 10.1186/s12864-022-08390-2 (PMC8882220; doi:10.1186/s12864-022-08390-2)

## Slide 1
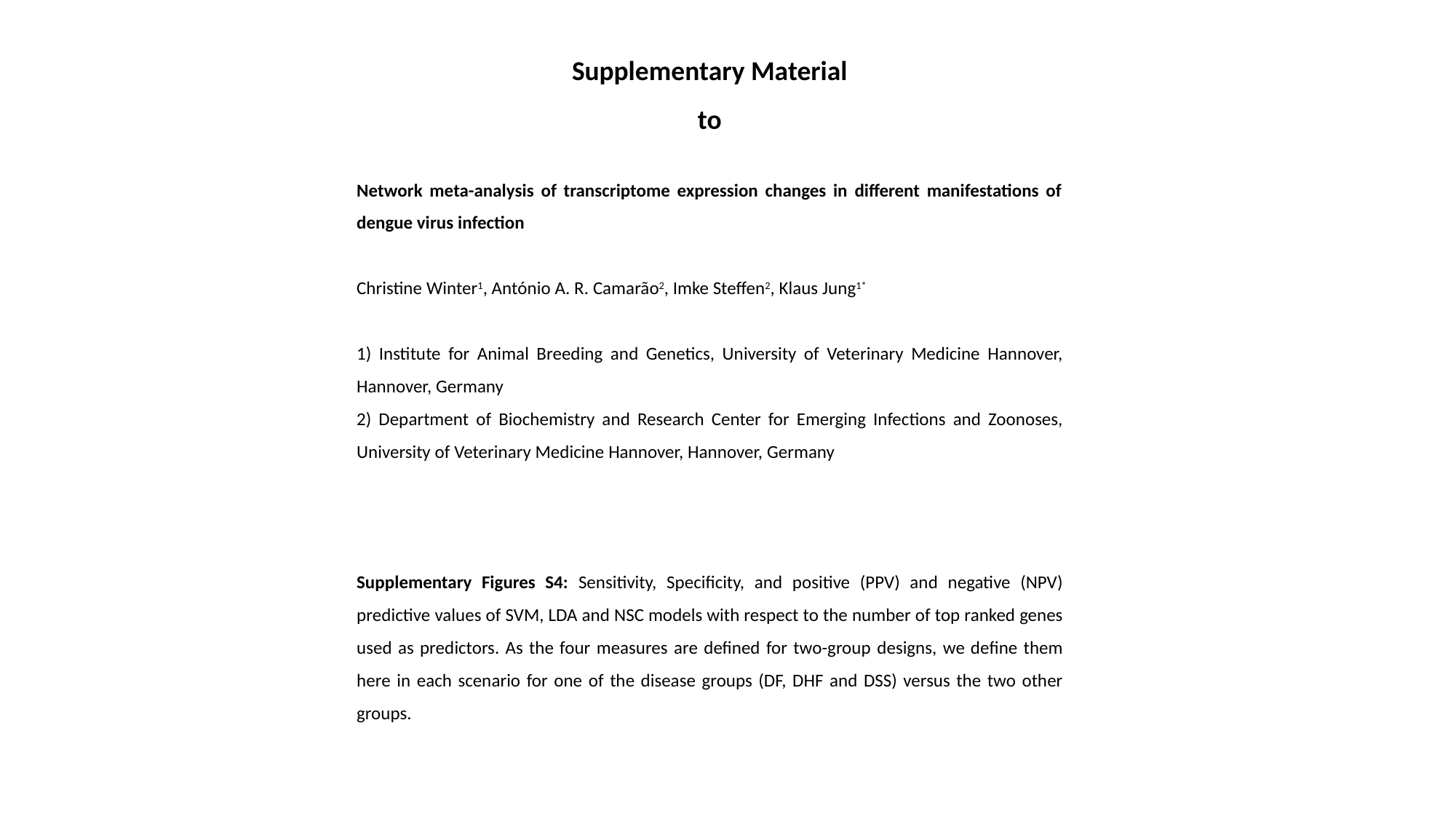

Supplementary Material
to
Network meta-analysis of transcriptome expression changes in different manifestations of dengue virus infection
Christine Winter1, António A. R. Camarão2, Imke Steffen2, Klaus Jung1*
1) Institute for Animal Breeding and Genetics, University of Veterinary Medicine Hannover, Hannover, Germany
2) Department of Biochemistry and Research Center for Emerging Infections and Zoonoses, University of Veterinary Medicine Hannover, Hannover, Germany
Supplementary Figures S4: Sensitivity, Specificity, and positive (PPV) and negative (NPV) predictive values of SVM, LDA and NSC models with respect to the number of top ranked genes used as predictors. As the four measures are defined for two-group designs, we define them here in each scenario for one of the disease groups (DF, DHF and DSS) versus the two other groups.

## Slide 2
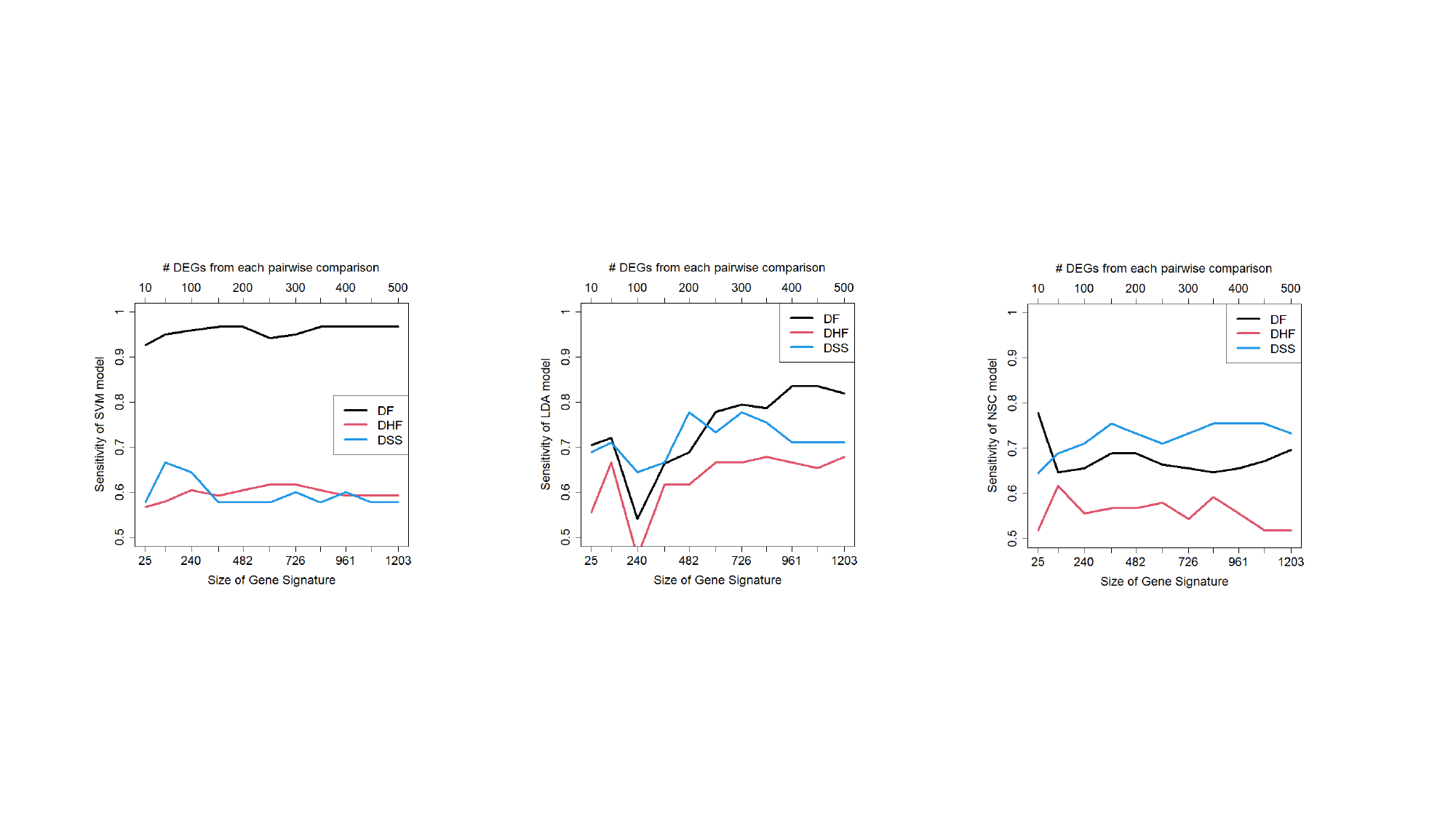

## Slide 3
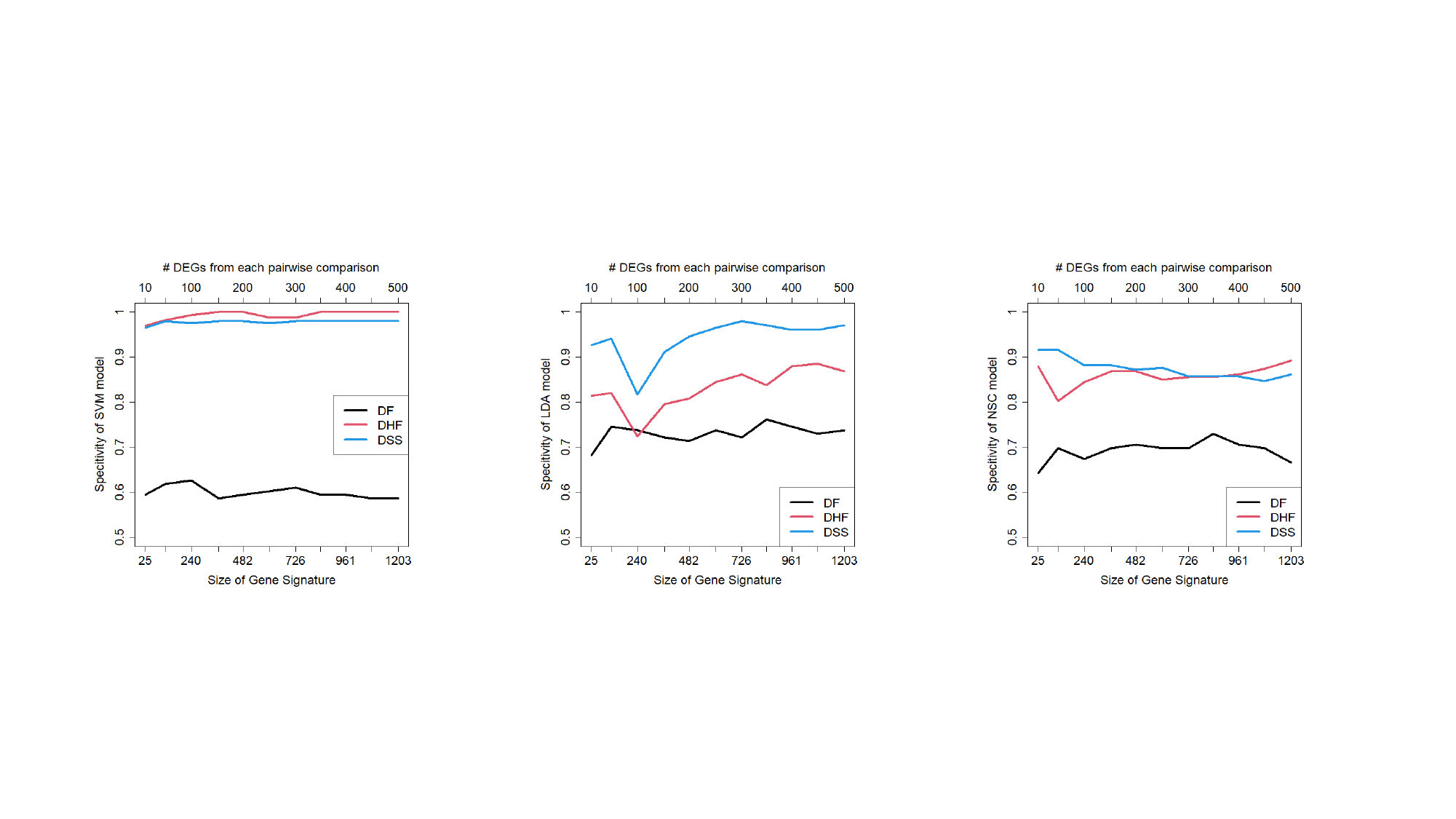

## Slide 4
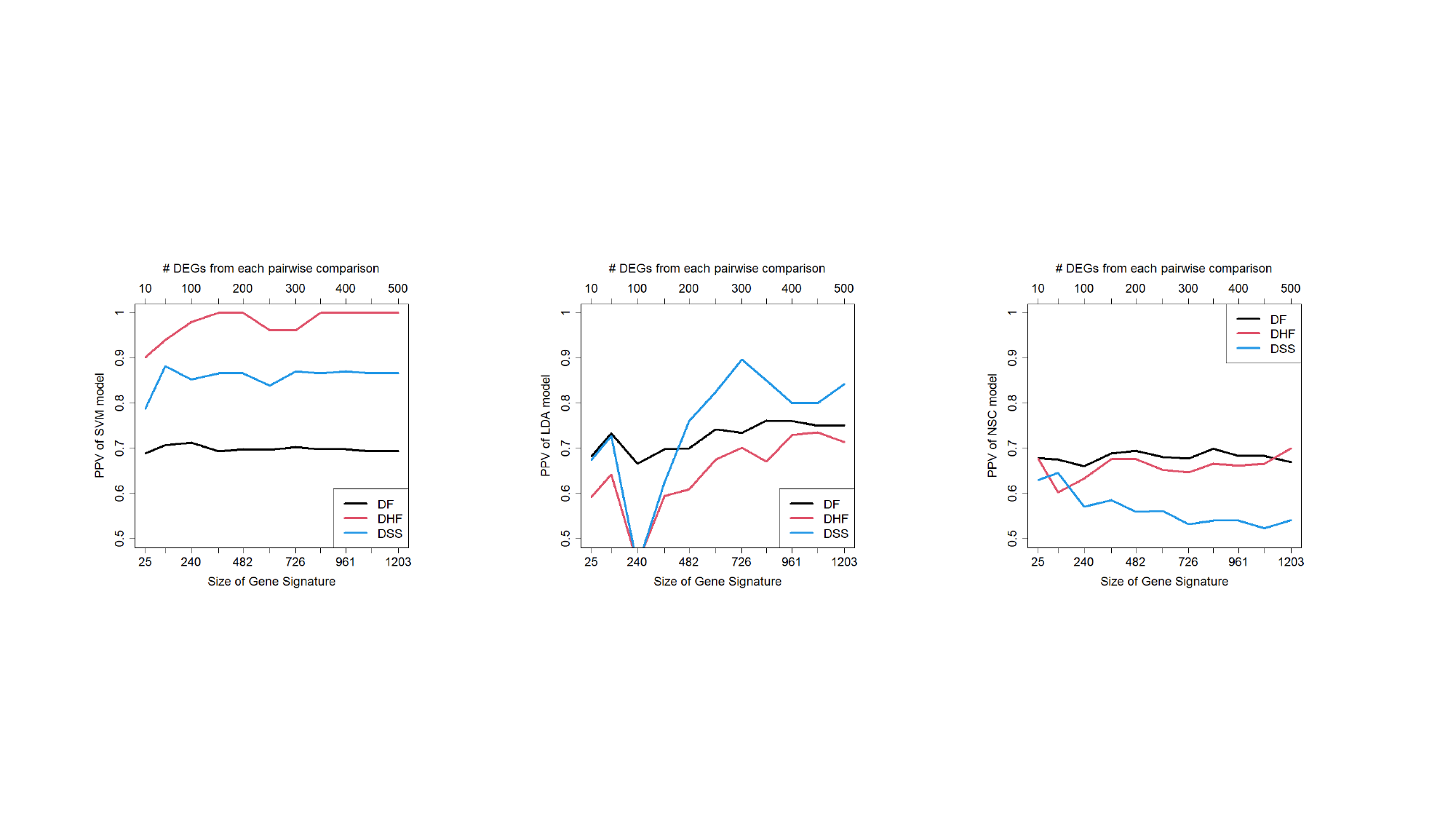

## Slide 5
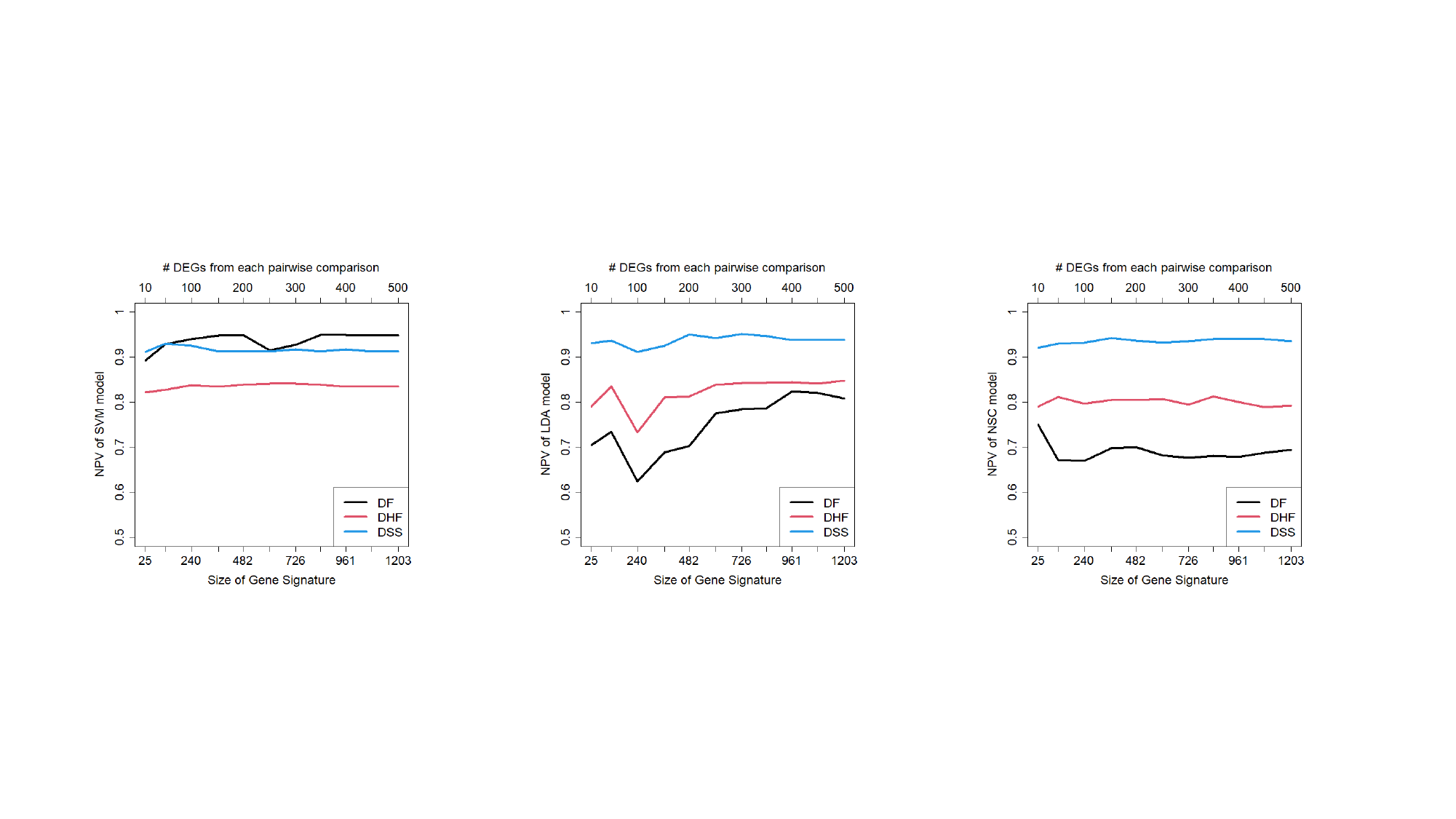

Supplement: Supplementary file 4 — Additional file 4: Supplementary Figure S4. Sensitivity, specificity, positive and negative predictive values of classifier models. [file 12864_2022_8390_MOESM4_ESM.pptx]
