# Supplementary material for: Network meta-analysis of transcriptome expression changes in different manifestations of dengue virus infection
Source: BMC Genomics. 2022 Feb 27;23:165. doi: 10.1186/s12864-022-08390-2 (PMC8882220; doi:10.1186/s12864-022-08390-2)
Supplement: Supplementary file 5 — Additional file 5: Supplementary Figure S5. Overlap of differentially expressed genes. [file 12864_2022_8390_MOESM5_ESM.pptx]

## Slide 1
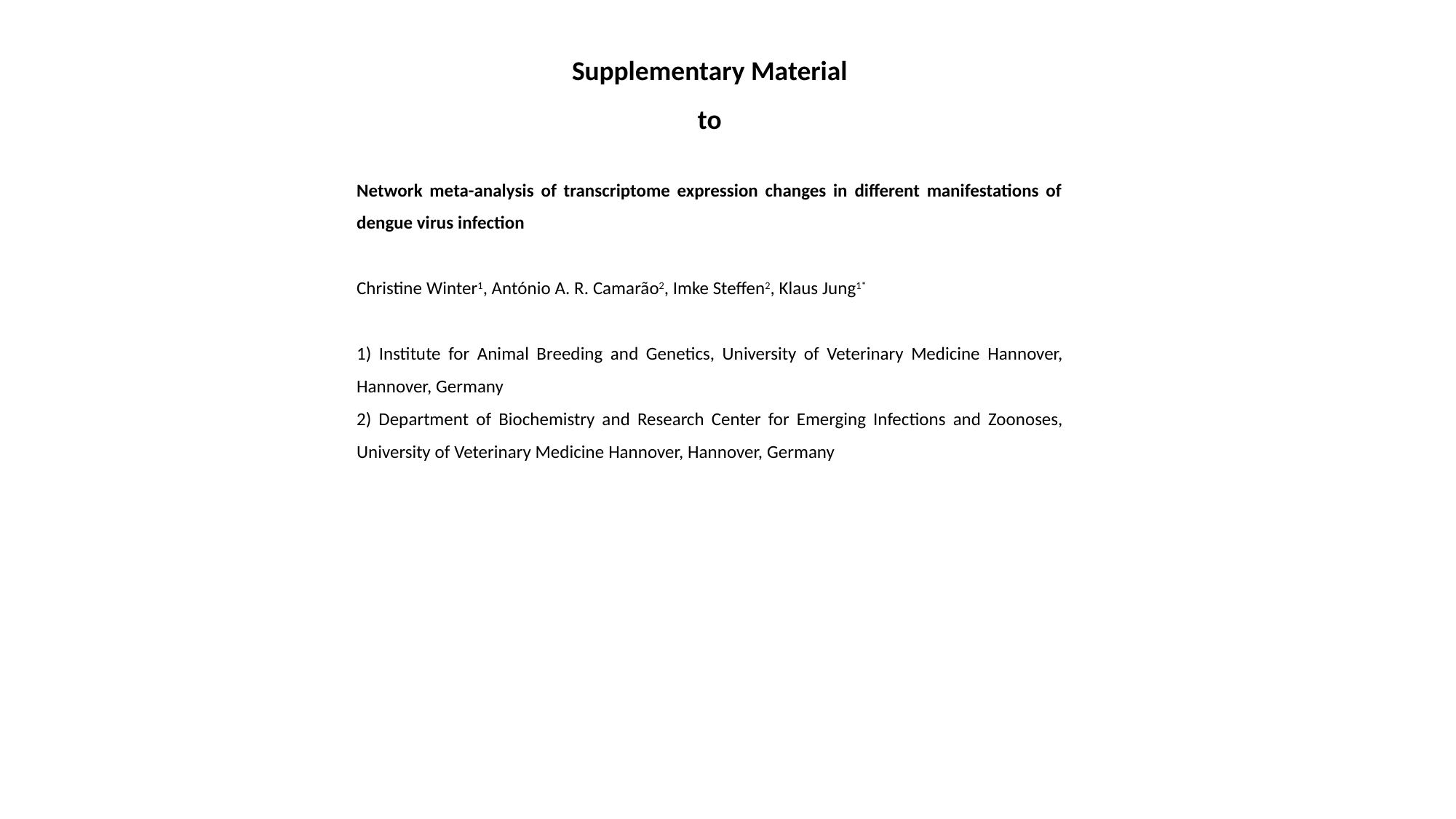

Supplementary Material
to
Network meta-analysis of transcriptome expression changes in different manifestations of dengue virus infection
Christine Winter1, António A. R. Camarão2, Imke Steffen2, Klaus Jung1*
1) Institute for Animal Breeding and Genetics, University of Veterinary Medicine Hannover, Hannover, Germany
2) Department of Biochemistry and Research Center for Emerging Infections and Zoonoses, University of Veterinary Medicine Hannover, Hannover, Germany

## Slide 2
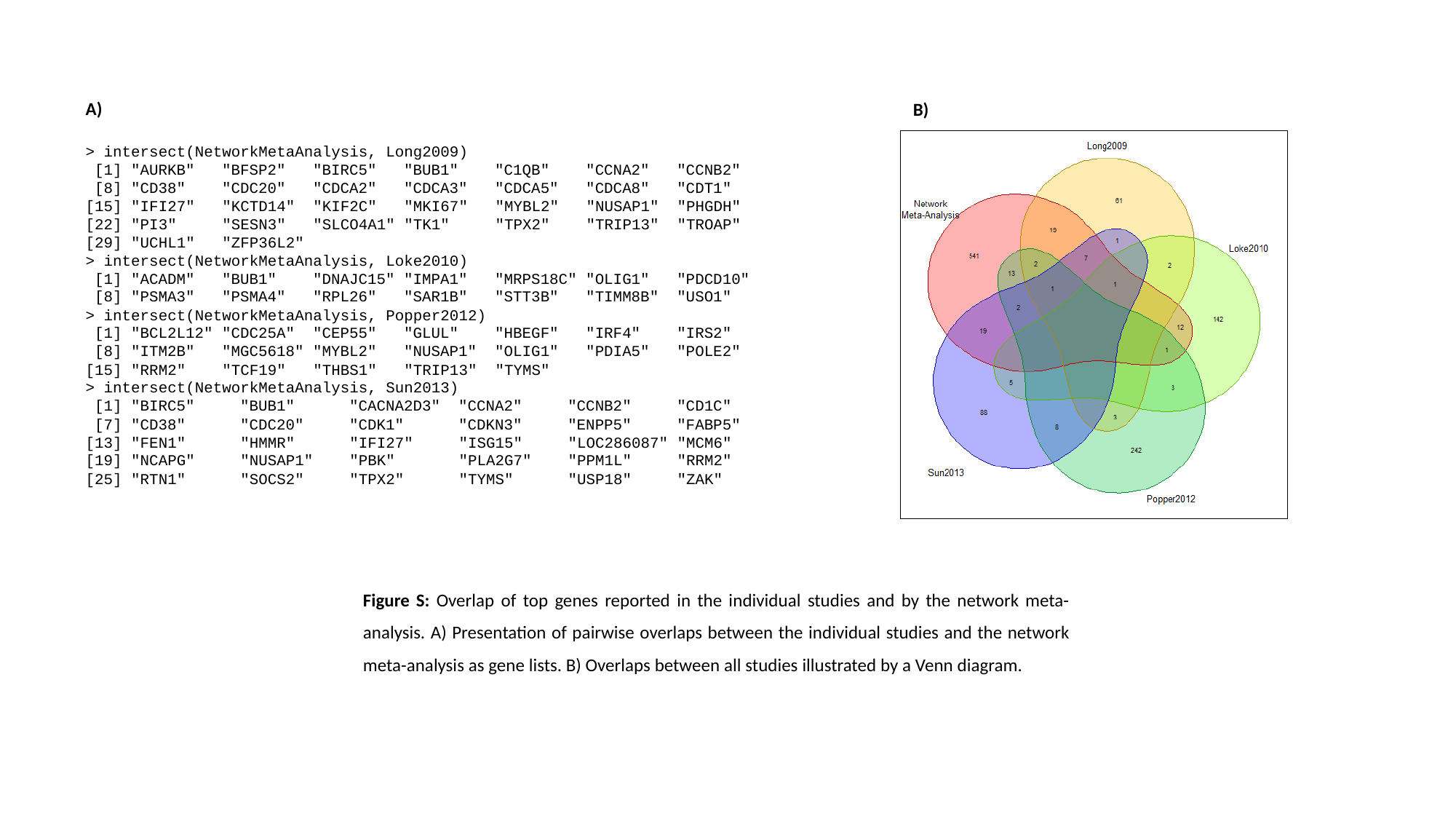

A)
B)
> intersect(NetworkMetaAnalysis, Long2009)
 [1] "AURKB" "BFSP2" "BIRC5" "BUB1" "C1QB" "CCNA2" "CCNB2"
 [8] "CD38" "CDC20" "CDCA2" "CDCA3" "CDCA5" "CDCA8" "CDT1"
[15] "IFI27" "KCTD14" "KIF2C" "MKI67" "MYBL2" "NUSAP1" "PHGDH"
[22] "PI3" "SESN3" "SLCO4A1" "TK1" "TPX2" "TRIP13" "TROAP"
[29] "UCHL1" "ZFP36L2"
> intersect(NetworkMetaAnalysis, Loke2010)
 [1] "ACADM" "BUB1" "DNAJC15" "IMPA1" "MRPS18C" "OLIG1" "PDCD10"
 [8] "PSMA3" "PSMA4" "RPL26" "SAR1B" "STT3B" "TIMM8B" "USO1"
> intersect(NetworkMetaAnalysis, Popper2012)
 [1] "BCL2L12" "CDC25A" "CEP55" "GLUL" "HBEGF" "IRF4" "IRS2"
 [8] "ITM2B" "MGC5618" "MYBL2" "NUSAP1" "OLIG1" "PDIA5" "POLE2"
[15] "RRM2" "TCF19" "THBS1" "TRIP13" "TYMS"
> intersect(NetworkMetaAnalysis, Sun2013)
 [1] "BIRC5" "BUB1" "CACNA2D3" "CCNA2" "CCNB2" "CD1C"
 [7] "CD38" "CDC20" "CDK1" "CDKN3" "ENPP5" "FABP5"
[13] "FEN1" "HMMR" "IFI27" "ISG15" "LOC286087" "MCM6"
[19] "NCAPG" "NUSAP1" "PBK" "PLA2G7" "PPM1L" "RRM2"
[25] "RTN1" "SOCS2" "TPX2" "TYMS" "USP18" "ZAK"
Figure S: Overlap of top genes reported in the individual studies and by the network meta-analysis. A) Presentation of pairwise overlaps between the individual studies and the network meta-analysis as gene lists. B) Overlaps between all studies illustrated by a Venn diagram.
