# Supplementary material for: Network meta-analysis of transcriptome expression changes in different manifestations of dengue virus infection
Source: BMC Genomics. 2022 Feb 27;23:165. doi: 10.1186/s12864-022-08390-2 (PMC8882220; doi:10.1186/s12864-022-08390-2)
Supplement: Supplementary file 8 — Additional file 8: Supplementary Table T3. Selection of 48 genes that occur frequently among the top 50, top 20, or top 10 of differentially expressed genes in the ten pairwise comparisons of the study network. The number give the absolute frequency, a gene occurs in one of the ten lists. [file 12864_2022_8390_MOESM8_ESM.docx]

| **Gene.ID** | **times among top 50** | **times among top 20** | **times among top 10** |
| --- | --- | --- | --- |
| IFI27 | 9 | 9 | 7 |
| TPX2 | 9 | 7 | 6 |
| DTL | 9 | 9 | 5 |
| KCTD14 | 9 | 9 | 5 |
| CDT1 | 10 | 6 | 5 |
| CDCA3 | 10 | 9 | 4 |
| CDC20 | 5 | 5 |  |
| CACNA2D3 | 7 | 4 |  |
| MCM10 | 7 | 4 |  |
| CDCA5 | 6 | 4 |  |
| HJURP | 6 | 4 |  |
| NCAPG | 6 | 4 |  |
| MKI67 | 5 | 4 |  |
| MRPS18A | 5 | 4 |  |
| CEP55 | 4 | 4 |  |
| MRPL22 | 4 | 4 |  |
| RNF141 | 4 | 4 |  |
| TK1 | 4 | 4 |  |
| CDCA2 | 8 |  |  |
| KIF2C | 8 |  |  |
| CCNA2 | 7 |  |  |
| POLE2 | 7 |  |  |
| RAD51 | 7 |  |  |
| TIGD3 | 7 |  |  |
| BIRC5 | 6 |  |  |
| CDC25A | 6 |  |  |
| CDC45 | 6 |  |  |
| ESPL1 | 6 |  |  |
| TTK | 6 |  |  |
| BAK1 | 5 |  |  |
| BARD1 | 5 |  |  |
| C16orf59 | 5 |  |  |
| C1QB | 5 |  |  |
| CENPA | 5 |  |  |
| FEN1 | 5 |  |  |
| OLIG1 | 5 |  |  |
| POC1A | 5 |  |  |
| TRIP13 | 5 |  |  |
| UBE2S | 5 |  |  |
| ATP2B1 | 4 |  |  |
| CD1C | 4 |  |  |
| CHEK1 | 4 |  |  |
| CNTNAP3 | 4 |  |  |
| DCPS | 4 |  |  |
| LDLR | 4 |  |  |
| LOC283588 | 4 |  |  |
| LOC286052 | 4 |  |  |
| PSME2 | 4 |  |  |

**Supplementary Table T3:** Selection of 48 genes that occur frequently among the top 50, top 20, or top 10 of differentially expressed genes in the ten pairwise comparisons of the study network. The number give the absolute frequency, a gene occurs in one of the ten lists.
